# Millimetre‐Scale Stratification of Microbial Communities in Hydrothermal Sediments

**DOI:** 10.1111/1462-2920.70227

**Published:** 2026-01-04

**Authors:** Janina Groninga, Weimin Liu, Lars Wörmer, Jenny Altun, Andreas Teske, Kai‐Uwe Hinrichs

**Affiliations:** ^1^ MARUM—Center for Marine Environmental Sciences University of Bremen Bremen Germany; ^2^ Faculty of Geosciences University of Bremen Bremen Germany; ^3^ Department of Earth, Marine and Environmental Sciences University of North Carolina at Chapel Hill Chapel Hill North Carolina USA

**Keywords:** anaerobic methane oxidation, Guaymas Basin, high‐resolution MS, intact polar lipids, MALDI‐FTICR‐MS, redox gradients

## Abstract

Resolving the spatial organisation of microbial populations in environments shaped by steep thermal and geochemical gradients remains a challenge in environmental biogeochemistry. Conventional molecular biomarker or gene‐based approaches typically require large volumes of homogenised samples, limiting their ability to depict spatially structured microbial ecosystems, where critical microbial processes occur on millimetre scales. To overcome these limitations, we applied high‐resolution mass spectrometry imaging (MSI) to an 11.5 cm long sediment section from the hydrothermal Cathedral Hill mat complex in the Guaymas Basin, known for its extreme temperatures and sharp geochemical gradients. The μm‐scaled spatial resolution unveiled a nuanced lipidome zonation tightly compressed to a narrow 5‐cm segment below the sediment–water interface. The surface layer (above 1.1 cmbsf) hosts molecular patterns primarily shaped by opposing oxygen and sulphide gradients, followed by a near‐seamless transition to an anoxic zone dominated by anaerobic methane‐oxidising archaea (ANME) and sulphate‐reducing bacteria (SRB). At greater depth, molecular signals indicative of active microbial communities remained below the detection limit except for diverse, potentially ANME‐ and SRB‐related lipids concentrated within a siliceous concretion. The sharp transitions in lipid zonation hint at persistent redox zones and resilient microbial niches under intense fluid flow and dynamic geochemical gradients.

## Graphical abstract

Zooming into the millimetre‐scale spatial distribution of organic molecules in hydrothermally impacted sediments using mass spectrometry imaging reveals a distinct lipid zonation with abrupt transitions at critical redox interfaces. This zonation reflects a pronounced stratification of diverse microbial communities and highlights surprisingly persistent microbial niches in this dynamic environment.

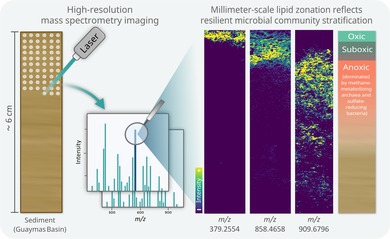

## 1 Introduction

Hydrothermal sediments are highly dynamic systems, shaped by intense fluid flow and steep physicochemical gradients unfolding on millimetre scales (Teske et al. 2016; Gundersen et al. 1992; Winkel et al. 2014). Together, these conditions create a complex biogeochemical habitat that supports diverse microbial populations and fosters the development of distinct ecological niches. However, resolving the fine‐scale spatial organisation of these microbial communities remains a challenge. Conventional gene‐based and molecular biomarker approaches typically rely on homogenised centimetre‐sized samples (Teske et al. 2002; Schouten et al. 2003; Bentley et al. 2022; Mara et al. 2022; Ramírez et al. 2021) that obscure the subtle spatial distribution of microbial communities that may occur across steep geochemical gradients. Consequently, it remains unclear whether steep redox gradients in hydrothermal environments promote a prominent depth‐stratification of different microbial populations or if any microbial organisation is disturbed by the intense and fluctuating fluid flow, leading to a more homogeneous distribution where functionally diverse microbes may coexist.

Here, we use hydrothermal sediments from the Guaymas Basin, located within the Gulf of California, as a natural laboratory for high‐resolution studies (Teske 2020). This young rift basin is shaped by intense geothermal activity and heat flux driven by the emplacement of hot doleritic sills into the sedimentary matrix (Einsele et al. 1980). The combination of the extreme heat and an up to 500‐m thick organic‐rich sediment cover (De La Lanza‐Espino and Soto 1999), fed by high oceanic productivity and substantial terrestrial input (Calvert 1966), creates a unique thermal and geochemical environment, where hydrothermal fluids are exceptionally enriched in methane, hydrocarbons, organic acids, hydrogen, sulphide and ammonia (Simoneit and Lonsdale 1982; Von Damm et al. 1985; Bazylinski et al. 1988; Martens 1990). In interaction with seawater‐derived electron acceptors such as oxygen, nitrate or sulphate, these strongly reducing hydrothermal fluids sustain a broad spectrum of metabolically diverse microbial populations within the surface sediments (Teske 2020; Teske et al. 2002; Dowell et al. 2016; McKay et al. 2016). As a result of steep redox and temperature gradients across millimetre‐to‐centimetre scales (Gundersen et al. 1992; Teske et al. 2016; Engelen et al. 2021), diverse mesophilic and thermophilic microbial communities driving processes such as aerobic sulphur oxidation, sulphate reduction, methanogenesis and anaerobic oxidation of methane (AOM) (Teske et al. 2002; McKay et al. 2016, 2012; Engelen et al. 2021) are compressed into a narrow near‐surface zone. By utilising Denaturing Gradient Gel Electrophoresis (DGGE) at 2 mm intervals, Engelen et al. (2021) highlighted gradually changing microbial communities with an evident coexistence of divergent metabolisms, including aerobic ammonia oxidation, sulphate reduction, anaerobic methane oxidation and fermentation. Such high‐resolution analyses are integral for accurately capturing microbial community distributions and their associated biogeochemical niches.

In contrast, conventional lipid biomarker studies in the Guaymas Basin typically relied on homogenised samples collected over centimetre intervals (Teske et al. 2002; Schouten et al. 2003; Bentley et al. 2022; Mara et al. 2022), limiting their ability to resolve any fine‐scaled shifts in microbial communities. To overcome these constraints in spatial resolution, an advanced technique, known as matrix‐assisted laser desorption ionisation mass spectrometry imaging (MALDI‐MSI), capable of resolving molecular distributions at much finer scales, has been adopted in environmental sciences (Wörmer et al. 2022; Alfken et al. 2020; Obreht et al. 2022). MALDI‐MSI can unlock previously inaccessible information about the fine‐scale spatial distribution of biomolecules by generating mass spectra from micrometre‐sized spots on undisturbed samples (Zenobi and Knochenmuss 1998; Knochenmuss 2006). In the past decade, MALDI‐MSI provided insight into biomarker localization in samples ranging from finely layered microbial mats (Wörmer et al. 2020) to laminated marine sediments (Obreht et al. 2022; Alfken et al. 2019), where it demonstrated great utility in leveraging biomarker‐based proxies for reconstruction of past environmental conditions with unparalleled temporal resolution.

In this study, we applied an untargeted MALDI‐MSI workflow, following an approach described by Liu et al. (2022), to capture a broad spectrum of lipid biomarker signatures in a sediment core retrieved from the hydrothermally active Cathedral Hill mat complex of the Guaymas Basin. By utilising non‐negative matrix factorisation (NMF) (Xiong et al. 2012; Leuschner et al. 2019; Nijs et al. 2021), we aimed to identify molecular signals with distinct spatial patterns reflecting microbial community stratification along the steep redox gradients characteristic of this hydrothermal environment. Following this approach, we provide an unprecedented insight into the redox‐dependent stratification of metabolically distinct microbial communities under extreme thermal and geochemical gradients.

## 2 Materials & Methods

### 2.1 Study Site and Geochemical Analysis

Sediment core 5000‐9 was retrieved during *Alvin* dive 5000 to the Cathedral Hill mat complex as part of the AT42‐05 deployment (Nov. 15–29, 2018) at the southern Guaymas Basin spreading centre. The core was taken from a hydrothermal hotspot, featuring intense overgrowth by orange *Beggiatoaceae* mats. Adjacent one‐point T‐sensor temperature measurements revealed a thermal gradient ranging from 33°C at 5 cmbsf to 69°C at 10 cmbsf and finally up to 86°C at 20 cmbsf (Ramírez et al. 2021). After recovery, the core was frozen and stored at −20°C until further analysis. A parallel core (5000‐3) taken within ~20 cm distance was used for oxygen, H_2_S and pH microsensor measurements, performed on the ship within 2 h after recovery following De Beer et al. (2006).

Total organic carbon (TOC), total nitrogen (TN), stable carbon and nitrogen isotopic composition (δ^13^C and δ^15^N) were determined from core 5000‐9 in 1 cm intervals. Approximately 0.5 g of sediment per interval was decalcified by treatment with 10% HCl and weighed into a tin capsule for duplicate analysis on a Flash 2000 organic elemental analyser (EA) connected to a Thermo Delta V Plus (Thermo Fisher Scientific GmbH, Bremen, Germany). δ^13^C and δ^15^N are expressed in ‰ relative to the Vienna Pee Dee Belemnite (VPDB) standard and atmospheric nitrogen. The resulting data are reported in Table S2.

### 2.2 Sample Preparation for MALDI‐MSI

The preparation of sediment samples for MALDI‐MSI typically involves sampling of wet sediment with LL‐Channels (Alfken et al. 2019). This approach was not feasible, as the frozen sediment core had a high water content and thawing risked disintegration, compromising the sediment integrity necessary for MALDI‐MSI. Instead, the frozen core was split using a sanitised saw. Two parallel sediment sections (width: ~1.5 cm; height: ~1 cm) were cut along the total core length, mimicking the dimensions of LL‐Channel sampling. To minimise thawing during preparation, the core was sampled at 4°C. One section was dedicated to conventional lipid biomarker analysis, aiding in validating and annotating MALDI‐MSI data. The sample, designated for MALDI‐MSI, was cut into two sections with the following dimensions (length × width × height): ~6.1 cm × 1.5 cm × 1 cm (5000‐9; 0–6.1 cmbsf) and ~5.4 cm × 1.5 cm × 1 cm (5000–9; 6.1–11.5 cmbsf).

The individual sediment sections were freeze‐dried and carefully embedded in a 5% gelatin and 2% carboxymethyl cellulose (CMC) mixture, following the method described by Alfken et al. (2019). For embedding, the sediment sections were placed into a silicon mould, while the gelatin/CMC solution was prepared by dissolving the components in deionised water (Milli‐Q) at ~60°C. The gelatin/CMC medium was added incrementally to the silicon mould until the sediment was completely covered. The embedded samples were frozen at −20°C for cryosectioning, where 100 μm‐thick sediment slices were cut using a cryomicrotome (−18°C; Microm HM 505 E Cryostat, GMI, Ramsey, Minnesota, USA). The sediment slices were mounted onto an indium tin oxide (ITO)‐coated glass slide (75 × 25 mm, Bruker Daltonik, Bremen, Germany) and adhered to its surface by warming the bottom of the slide.

### 2.3 Matrix Application

We chose two experimental approaches for MSI, each assisted by a different MALDI matrix to cover a broad range of compound classes. The matrices consisted of 10 mg m L^−1^ 1,5‐DAN (1,5‐diaminonaphtalene; ≥ 97%, Sigma‐Aldrich) and 10 mg m L^−1^ 2,5‐DHB (2,5‐dihydroxybenzoic acid; ≥ 99%, Sigma‐Aldrich), both dissolved in 70:30 ACN/H_2_O (acetonitrile/water) and were applied using the HTX TM‐Sprayer (HTX Technologies, Chapel Hill NC, USA). The sprayer deposited a matrix aerosol onto the sample within an enclosed, nitrogen‐flooded chamber. Spray coating was performed using the following parameters: HTX temperature of 75°C, a velocity of 1200 mm m in^−1^, flow rate of 0.12 mL m in^−1^ and a 2‐s drying time. For 2,5‐DHB coating, 30 layers were applied, achieving a matrix density of 0.01 mg m^−2^. For 1,5‐DAN, the number of layers was reduced to eight, corresponding to a matrix density of 0.0027 mg m^−2^. The coated samples were measured within 24 h of matrix application to prevent matrix degradation and to ensure optimum ionisation.

### 2.4 MALDI‐MSI

MSI of molecular biomarkers was performed on a 7T solariX XR FTICR‐MS coupled to a MALDI source with a Smartbeam II laser (Bruker Daltonik, Bremen, Germany). Before measurement, external mass calibration was performed in positive and negative polarity in electrospray ionisation (ESI) using sodium trifluoroacetate (NaTFA; Sigma‐Aldrich), according to Moini et al. (1998). MS data were acquired within an analytical mass window of *m/z* 150 to 1000. All measurements were conducted in CASI (continuous accumulation of selected ions) mode. Since narrower CASI windows typically improve measurement sensitivity and increase the signal‐to‐noise ratio (SNR) (Wörmer et al. 2019; Alfken et al. 2019), analyte detection was divided into five CASI windows comprising the following *m/z* ranges: 150–350; 310–460; 460–610; 600–800; and 800–1000. To cover the analytical *m/z* window for each MALDI matrix (1,5‐DAN and 2,5‐DHB), we performed measurements on six sediment slices. Specifically, three sediment slices were used per MALDI‐Matrix, each slice comprising one or two CASI windows per measurement region, utilising the alternating spot method. The alternating spot aims to maximise data generation from the same measurement area, bypassing the destructive nature of MALDI, and operates as follows: By using a medium laser size (75 μm) and a spot distance of 150 μm, the same measurement could be used for two CASI windows by applying a spatial offset of 75 μm.

MALDI‐MSI assisted by a 1,5‐DAN matrix was performed in negative polarity, while 2,5‐DHB‐coated samples were measured in positive ionisation mode. The laser settings were adjusted to acquire optimal signal intensities and ensure a total ion count (TIC) within a range of 1 × 10^8^ and 1 × 10^9^ counts to minimise ion suppression, overall resulting in a laser power between 60% and 90%, the number of laser shots ranging from 250 to 700, and a laser frequency between 250 and 700 Hz. Laser settings varied depending on the *m/z* range, the respective matrix coating and specific values for each measurement are presented in Table S1. Time‐of‐flight was adjusted according to the *m/z* range, with values of 0.5–0.7 s chosen for the lower *m/z* range (150 to 600) and 0.8–1.1 s set for the higher mass range (600 to 1000). Similarly, the collision voltage was set to −5 V in positive ion mode or + 5 V in negative ion mode for the *m/z* range below 600 and −10 V (positive ion mode) and + 10 V (negative ion mode) for the *m/z* range above 600. No data reduction was applied.

MALDI‐MSI was performed using FlexImaging (Bruker Daltonik, Bremen, Germany), generating 15,953 to 18,864 individual spectra per analysis, depending on the dimensions of the individual sediment slices. Initial data preprocessing in DataAnalysis 5.0 (Bruker Daltonik, Bremen, Germany) consisted of lock‐mass calibration of raw spectra and SNR filtering with a threshold of 4 to exclude instrumental noise. We opted to lower the commonly applied SNR of 5 to retain potentially meaningful low‐intensity signals, justified by additional noise reduction steps in subsequent peak picking that would exclude instrumental noise from downstream evaluation. Internal lock‐mass calibration was performed using either calculated *m/z* values of matrix oligomers (Treu and Römpp 2021) or known compounds within the respective CASI windows. A detailed listing of chosen calibrants is given in Table S1. The SNR‐filtered and lock‐mass‐calibrated mass spectra were then exported and stored in comma‐separated plain text files, including *m/z*, peak intensity and SNR for every *x*, *y* coordinate.

### 2.5 Untargeted Data Mining Workflow

MSI datasets in the form of plain text files were subsequently fed to an untargeted data mining workflow following Liu et al. (2022). In brief, peak intensities were first normalised by the median peak intensity of each spectrum. Bin‐wise kernel density estimation (KDE) was applied for peak *m/z* alignment, where only peaks with a prominence greater than 0.01 on the density curve were kept, removing potentially noisy signals. Additionally, molecular features with high sparsity, i.e., a high proportion of zero‐intensity values, over the sample region were excluded to reduce noise for downstream analysis. To calculate the sparsity of each feature, we defined spatial regions by binning × coordinates into zones with an interval of 30 units, followed by quantification of the fraction of non‐zero values for each feature therein. After numerous tests, a sparsity threshold of 7% was deemed adequate for retaining meaningful features in the subsequent NMF analysis.

### 2.6 Data Cleaning

The matrix coating introduces numerous high‐intensity ions unrelated to the sediment sample. To exclude these signals from downstream analysis, we leveraged the void regions within the measurement area that are remnants of the sample preparation process. During freezing, the porewater expanded, leaving small gaps within the sediment, which were later filled by the embedding mixtures and coated with the MALDI matrix. Accordingly, peaks whose intensity was disproportionately concentrated in the void regions within the sediment samples were excluded. To define the voids, we used high‐resolution TIFF images of the measurement area as visual indicators. We identified voids based on the pixel colour and by manual examination in FlexImaging (Figure S1). By matching the spatial coordinates of the feature table and the image pixels, we determined whether the intensity signal of a feature stems from the sediment or the void regions. A disproportionate presence within void regions resulted in exclusion from NMF decomposition.

### 2.7 Molecular Fingerprint Identification

NMF was subsequently applied to identify meaningful molecular signatures. In brief, NMF performs dimensionality reduction, which allows us to extract groups of co‐occurring ions that represent the underlying molecular patterns (components) within the MSI dataset. For noise reduction in preparation for NMF, the TIC‐normalised dataset was divided into 30‐unit spatial zones with the calculation of the average abundance for each *m/z* therein. Prior to applying NMF, the data was scaled using MaxAbsScaler, which normalised the feature intensities to [0, 1], ensuring equal contribution of all features to the NMF decomposition. To determine the optimal number of components for NMF, we iteratively minimised the reconstruction error until it stabilised below 0.0015. NMF then decomposed the dataset into a feature matrix that represents the contributions of each *m/z* to the components (pseudo spectra) and a spatial matrix describing the distribution of each component across the sample (molecular fingerprints). To visualise the spatial distribution of the identified components, a weighted average ion image was created for each component, where the pixel intensities are calculated by weighing the TIC‐normalised abundances of the features by their contributions to the component. To emphasise dominant features, the contributions of each component were raised to the fifth power. The feature matrix containing the contribution of each *m/z* to the components was exported and features that drive the biogeochemically meaningful patterns were selected.

### 2.8 Lipid Extraction and UHPLC‐ESI‐QTOF‐MS/MS

For lipid extraction, sediment was collected from push core 5000‐9, parallel to the section dedicated to MALDI‐MSI, offset by approximately 1 cm, to ensure comparability. Samples were taken at 2 cm depth intervals, yielding approximately 1 g of freeze‐dried sediment. Lipid extracts were prepared following the modified Bligh & Dyer protocol (Sturt et al. 2004) using a solvent mixture of dichloromethane:methanol:buffer (DCM:MeOH:buffer, 1:2:0.8, v:v:v) assisted by ultrasonication (10 min per step). The buffer consisted of monopotassium phosphate (8.7 g L^−1^, pH 7.4) for the first two extraction steps and trichloroacetic acid (50 g L^−1^, pH 2) for the final two extraction steps. The total lipid extracts (TLE) were dried under a gentle stream of nitrogen and stored at −20°C until further analysis.

Aliquots of the TLE were analysed via reversed‐phase ultra‐high‐performance liquid chromatography (RP‐UHPLC) coupled with electrospray ionisation tandem mass spectrometry (ESI‐MS/MS) utilising a Dionex Ultimate 3000 RS ultra‐high‐performance LC system following the method of Wörmer et al. (2013). Chromatographic separation was achieved using an Acquity UPLC BEH C18 column (1.7 μm; 2.1 × 150 mm, Waters Corporation, Eschborn, Germany), with the mobile phase consisting of eluent A (methanol: water; 85:15; 0.04% formic acid, 0.1% NH_4_OH) and eluent B (propan‐2‐ol:methanol; 50:50; 0.04% formic acid, 0.1% NH_4_OH). The flow rate was set to 0.4 mL m in^−1^ with the elution programme starting at 0% B, increasing to 15% B by 2 min and 85% B by 20 min, followed by an 8‐min column washing with 100% B. The column temperature was maintained at 65°C.

The RP‐UHPLC system was coupled to a Bruker maXis ultra‐high‐resolution quadrupole time‐of‐flight mass spectrometer (UHR‐qToF, Bruker Daltonics, Bremen, Germany) comprising an ESI interface. Spectral acquisition was conducted in positive ionisation across an *m/z* range of 50–2000 at a rate of 2 Hz. Mass calibration was achieved via loop injections of Agilent TuneMix calibration standard solution at the end of each run and further correction using a lock mass calibrant (992.0098), resulting in a mass accuracy better than 2 ppm. Lipid identification was based on retention time patterns, the accurate *m/z* and characteristic MS/MS fragmentation.

### 2.9 Micro X‐Ray Fluorescence Spectroscopy (μXRF)

Elemental mapping was performed on analogue 100 μm thick sediment slices (0–6.1 and 6.1–11.5 cmbsf) using an M4 Tornado system (Bruker Nano GmbH, Berlin, Germany) equipped with an Rh source (50 kV, 600 μA) with a poly‐capillary optic. μXRF spectra were acquired under a vacuum (20 mbar) in one cycle with 30 ms/pixel and a step size of 50 μm, yielding 409,740 pixels for the entire sediment section. Elemental contributions were deconvoluted and quantified for spatial mapping based on relative count intensities using M4 Tornado version 1.3.

## 3 Results and Discussion

### 3.1 Geochemical Contextualization of Cathedral Hill Sediment

The geochemical and thermal framework of the Cathedral Hill sediment core (5000‐9) provides a foundation for discussing redox‐dependent microbial activity and biogeochemical zonation in this unique environment. The Cathedral Hill mat complex in the Guaymas Basin has been a popular target for researching biogeochemical processes in hydrothermally active sites due to the exceptionally high organic matter content, abruptly shifting redox conditions and its extreme thermal regime (Teske et al. 2016; Ramírez et al. 2021; Su et al. 2023). Notably, TOC values reached up to 4.8% in the surficial sediment, remaining above 1.7% throughout the entire core (Table S2). One‐point temperature‐sensor measurements adjacent to core 5000‐9, starting from an assumed bottom water temperature of ~3°C at the sediment surface, revealed a steep thermal gradient, with temperature rising to 35°C at 5 cm below seafloor (cmbsf), 69°C at 10 cmbsf and 86°C at 20 cmbsf (Ramírez et al. 2021). Although sedimentation rates for Cathedral Hill are not available, published estimates for the Guaymas Basin generally range from approximately 0.2 mm to 1 mm year^−1^, indicating that the surficial sediment layers represent relatively young material (Teske et al. 2019).

**FIGURE 1:**
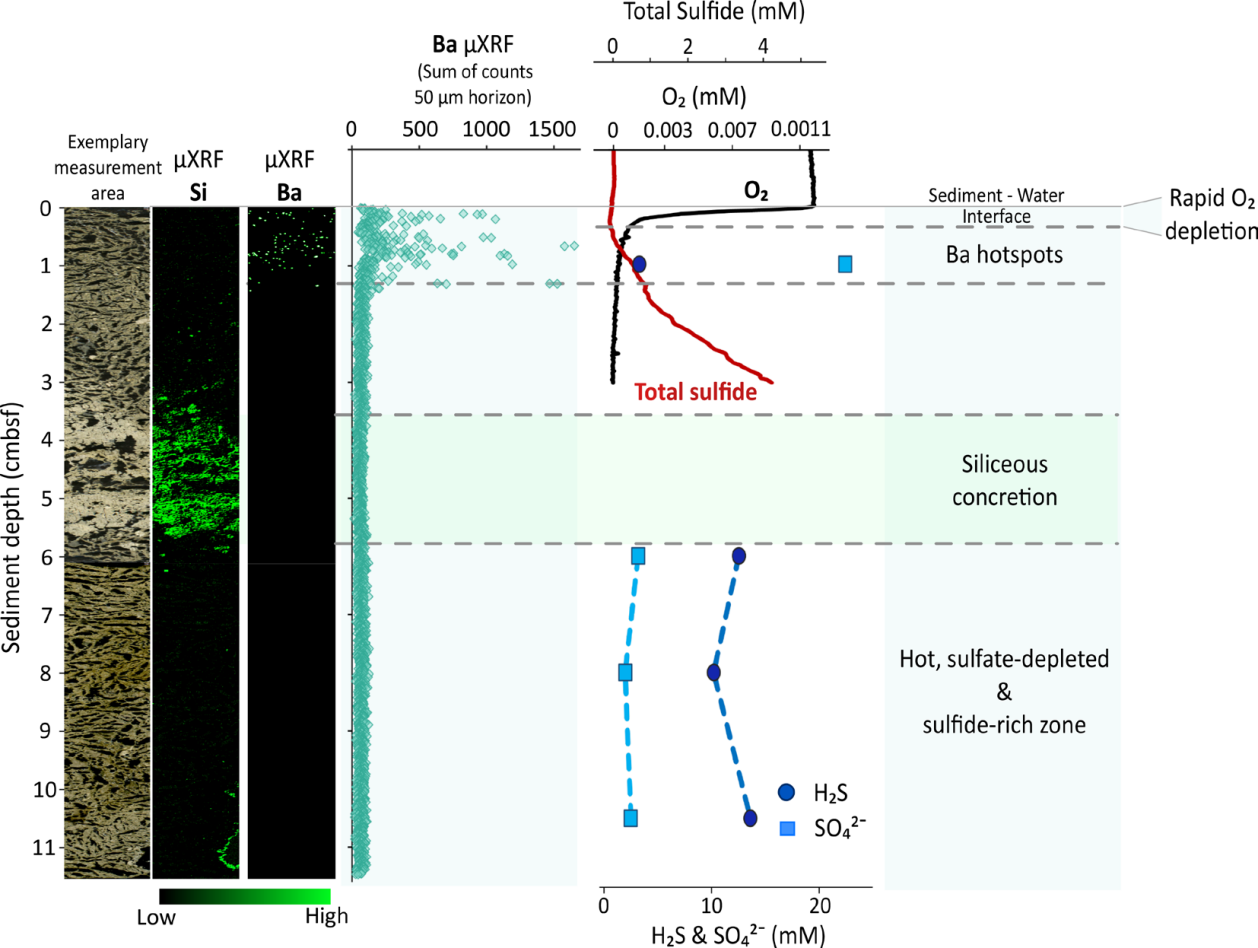
Geochemical zonation of Cathedral Hill sediment. Integration of multiple datasets to illustrate the geochemical conditions within the upper sediment column. On the left, μXRF elemental maps depict the spatial distribution of silicon (Si) and barium (Ba; μXRF—Lß emission line: 4.842 keV) in relative counts with a complementary intensity depth profile (sum of counts in 50 μm bins) of Ba to the right. Adjacent in situ oxygen profiles (mM), determined with microprofiler sensors at a step size of 250 μm according to De Beer et al. (2006) on core 5000‐3 are shown alongside porewater profiles of sulphate (SO_4_^2−^) and sulphide (H_2_S), both of which were determined via Rhizon sampling from neighbouring core 5000‐6, published in Ramírez et al. (2021).

The geochemical data illustrated in Figure 1 highlight the dynamic redox transitions within the sediment inferred from shipboard microprofiling (step size 250 μm) of dissolved oxygen in neighbouring core 5000‐3 (De Beer et al. 2006). O_2_ concentration declined to ~2 μM below the sediment–water interface, with low oxygen levels of 0.3–1 μM prevailing until ~1 cmbsf. This is consistent with prior finely resolved in situ microprofiler surveys conducted in the Guaymas Basin (Teske et al. 2016; Winkel et al. 2014). Complementary microprofiling of total sulphide (Figure 1) reveals a sharp upward decrease to non‐detectable levels in the surface zone above 1 cmbsf, indicative of active aerobic sulphide‐oxidising communities, likely including the abundant thiotrophic *Beggiatoa* colonising the sediment surface (Gundersen et al. 1992; Jannasch et al. 1989). Sulphide‐oxidising bacteria flourish even under low oxygen levels and have been observed to couple sulphide oxidation and nitrate reduction (MacGregor et al. 2013; Schutte et al. 2018). Within this surficial niche, sulphide oxidizers likely coexist with diverse aerobic microbial communities, including nitrifying bacteria and archaea (Engelen et al. 2021; Winkel et al. 2014) and aerobic methanotrophs (Teske et al. 2021). Moreover, localised oxygen intrusion through hydrothermal pumping, fueling aerobic sulphide oxidation, has been documented down to 1 cm into the sediment (Teske et al. 2016; Gundersen et al. 1992; Winkel et al. 2014). It must be noted that the presented geochemical and thermal framework represents a snapshot of conditions present during sample collection and substantial fluctuations in the advective flow of hydrothermal fluids may result in rapid microbial community shifts (Engelen et al. 2021; McKay et al. 2012).

A notable downcore decline of porewater SO_4_^2−^ concentrations to near depletion is evident within ~5 cmbsf, while H_2_S levels show a concurrent increase (Ramírez et al. 2021), indicative of dissimilatory sulphate reduction (Figure 1). Although porewater profiles were obtained from neighbouring sediment cores (Cores 5000‐3 and 5000‐6), their spatial proximity (within approximately 10–20 cm of core 5000‐9) allows a reasonable approximation of the prevailing conditions relevant to this study.

Cryosectioning of the embedded sediment revealed a single, discrete whitish concretion at approximately 3.5–5.5 cmbsf, which was of siliceous composition (Figure 1). Siliceous precipitates are not uncommon in the Guaymas Basin. For instance, at the Ringvent site, multiple amorphous silica (i.e., opal‐A) nodules were observed in subsurface sediment and attributed to the conductive cooling of silica‐saturated hydrothermal fluids combined with seawater mixing (Teske et al. 2019). This nodule might result from in situ precipitation within the subsurface sediment, or it could represent a buried fragment of a larger siliceous concretion from the surface.

Another element with conspicuous vertical distribution is barium, which displays a spotted pattern concentrated in the surficial sediment. Translated into a depth profile, barium remains near the detection limit almost throughout the entirety of the sediment column but shows high‐intensity hotspots located between 0 and 1 cmbsf. The dispersed distribution is consistent with the presence of finely distributed baryte (BaSO_4_) precipitates. In hydrothermal environments, baryte precipitation typically results from the interplay between ascending barium‐rich hydrothermal fluids and downwards diffusing sulphate‐rich seawater (Magenheim and Gieskes 1992; Torres et al. 1996), consistent with the observed high sulphate concentrations (> 20 mM) at the surface (Figure 1). Below ~1 cmbsf, the baryte is mobilised due to progressing sulphate depletion promoted by the intense activity of sulphate‐reducing bacteria (Bolze et al. 1974; McManus et al. 1998), essentially marking the onset of a stable reducing environment.

### 3.2 Molecular Fingerprints

**FIGURE 2:**
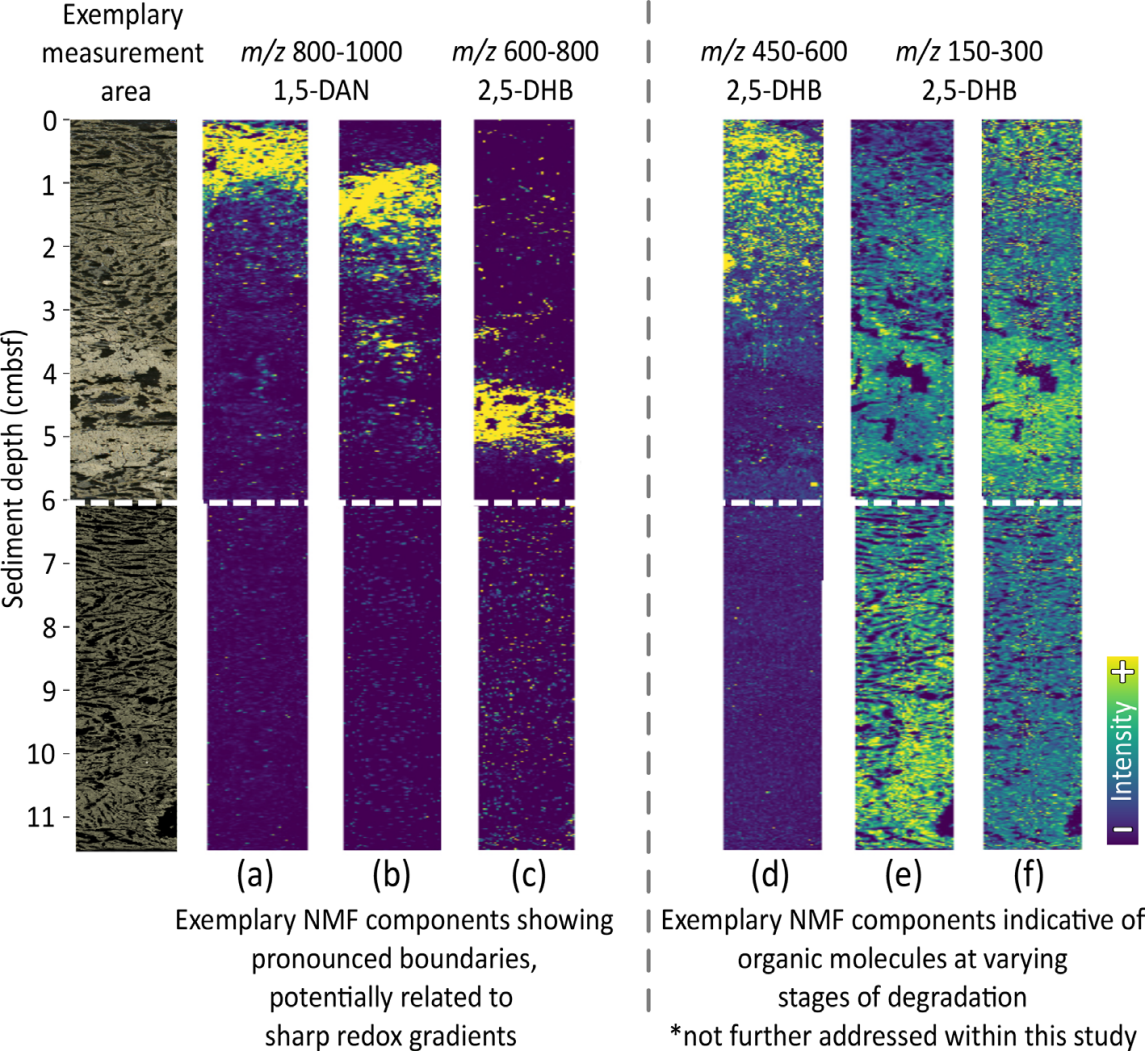
Measurement area and representative NMF components from MALDI‐MSI of Guaymas Basin sediment (core 5000‐9; 0–11.5 cmbsf). The left panel shows the measurement area on a sediment slice used for MALDI‐MSI. Adjacent (a–f) display representative average ion images derived from NMF analysis, each representing a spatially co‐occurring group of molecular features (NMF component). NMF analysis was performed separately for each matrix (1,5‐DAN and 2,5‐DHB) and measured *m/z* range (cf. Section 2 for details on *m/z* windows and matrix application).

MALDI‐MSI data were acquired in positive and negative ionisation modes using two different matrices (2,5‐DHB and 1,5‐DAN). To obtain comprehensive and high‐resolution molecular data, measurements were conducted over five *m/z* ranges using the CASI function. In total, 344,904 mass spectra were acquired from the 11.5‐cm‐long sediment section of core 5000‐9. From these, after peak picking, a combined dataset of 64,667 molecular features was obtained (Table S1). Subsequent data filtering, excluding matrix‐related signals and low‐abundance peaks, reduced the dataset to 11,819 features. NMF analysis then revealed distinct molecular distribution patterns, hereafter referred to as NMF components. These NMF components form the basis of the following discussion of biogeochemically meaningful molecular signatures across the downcore profile. The agreement between the NMF components and the biogeochemical zonation suggests a strong link between lipids and the environments in which they accumulated, whether due to biotic or abiotic processes. This assertion is supported by a selection of weighted average ion images showcasing spatially distinct NMF components (Figure 2a–f). In general, we observe two fundamentally different types of spatial patterns. First, several components feature sharp vertical transitions, reminiscent of the steep redox gradients typical of the hydrothermal sediment (Figure 2a–c). We interpret molecular distribution patterns characterised by these abrupt and seemingly reciprocal transitions (Figure 2a,b) to be indicators of localised, redox‐driven microbial activity within the sediment. By contrast, other molecular fingerprints show a more uniform distribution or gradual downward or upward trends (Figure 2d–f). These smoother patterns are more consistent with the progressive degradation of potentially water‐column‐derived signals or persisting recalcitrant organic molecules. For instance, many features that are most prominent at the surface and exhibit a smoother, gradual downward decreasing trend (Figure 2d) are annotated as molecular formulas that are consistent with degradation products of chlorophyll, including pyropheophorbide *a*, pyropheophytin *a* (Ziegler et al. 1988; Shioi et al. 1991), but also other tetrapyrroles such as heme and long‐chain polyamines, the latter being produced by diatoms in the high‐productivity surface water (Kröger et al. 2000) (Figure S2). Molecular features showing a uniform distribution or a downward‐increasing trend are predominantly associated with smaller molecules with *m/z* values below 500. These spatial patterns encompass hundreds to thousands of individual molecular features and are beyond the scope of this study.

### 3.3 Molecular Distribution Patterns in Surface Sediment

**FIGURE 3:**
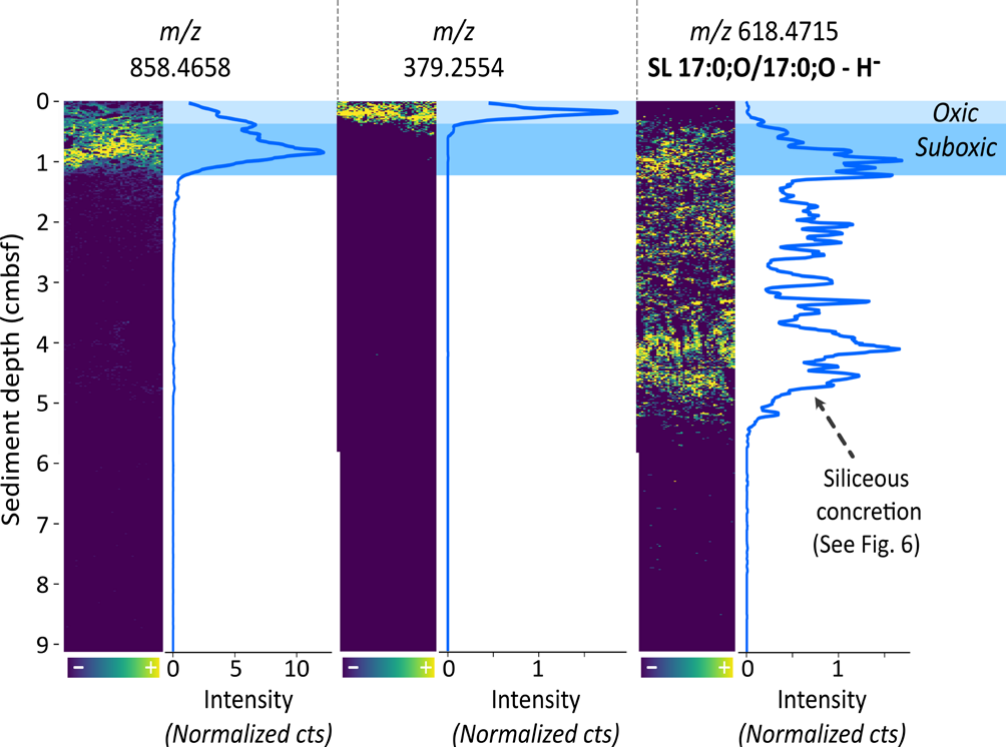
Spatial distribution of molecular features in surface sediment of the Guaymas Basin. Ion intensity plots for three selected molecular features detected via MALDI‐MSI in negative ion mode using a 1,5‐DAN matrix, showing distinct spatial patterns. Line plots adjacent to each heatmap illustrate the sum of normalised counts for each 150‐μm depth horizon, smoothed using a moving average of five data points. Colour gradients in the heatmaps represent normalised intensity, ranging from low intensity (blue) to high intensity (yellow). Overlaid blue and cyan bars indicate the redox stratification of the sediment, with oxic and suboxic zones labelled for reference. Sulfonolipid (SL): 17:0;O/17:0;O represents the fatty acid composition of the lipid with 17 and 0 indicating the number of carbon atoms and double bonds in one hydrocarbon chain respectively and O specifying the presence of a hydroxyl group in the acyl moiety.

To investigate molecular signals presumably linked to oxygen availability, we extracted features spatially concentrated near the surface (Figure 2a). Among these, we selected three ions with comparably high peak intensities that exemplify the distinct molecular patterns observed within the surface zone (Figure 3).

The molecular ion *m/z* 858.4658 (Figure 3) gradually increases until reaching the maximum intensity within the suboxic zone and then instantly disappears at 1.1 cm depth. Additional ions sharing this distribution are shown in Figure S3. These distinct molecular fingerprints are consistent with the diffusion of seawater‐derived electron acceptors such as oxygen and nitrate into surficial sediments. Although we cannot determine a definitive source of these compounds, these conditions are generally favourable for aerobic and nitrate‐reducing microbial communities involved in the oxidation of sulphide (see also sulphide profile in Figure 1), nitrate/nitrite and methane (Teske et al. 2021; Engelen et al. 2021). Interestingly, the disappearance of *m/z* 858.4658 spatially coincides with that of barium (Figure 1), which we hypothesise to be driven by intense microbial sulphate reduction, effectively marking the onset of a stable reducing, anoxic environment at 1.1 cmbsf. Below, O_2_ and nitrate are no longer available, and redox‐sensitive microbial communities dependent on these terminal electron acceptors do not persist. While the precise sources of *m/z* 858.4658 and other compounds sharing the same near‐surface distribution (Figure S3) remain unidentified, their sharp, depth‐restricted occurrence is consistent with oxygen and/or nitrate availability and links them to active redox‐driven processes near the sediment–water interface.

The surface sediment features a remarkably narrow zone, just 0.2–0.3 cm below the sediment–water interface, hosting several unidentified compounds best represented by *m/z* 379.2554 (Figures 3 and S3). These molecules are located in the zone characterised by the rapid decline of oxygen just millimetres below the surface (Figure 1), implying strictly aerobic microbial taxa as the potential source. Although a particular source organism cannot be identified and we cannot exclude external input settling on the sediment surface, the sharp, depth‐restricted spatial organisation is more consistent with microbial processes governed by steep redox gradients in the surface sediment. This spatial pattern is further emphasised by the near‐complementary appearance of *m/z* 618.4715 at 2–3 mm depth, from where it extends several centimetres deeper into the reducing zone (Figure 3). Additionally, this compound coincides with the silica concretion between ~3.5 and 5.5 cmbsf. Complementary UHPLC/HRMS lipid data obtained from a corresponding sediment sample suggested that the compound is a sulphonolipid (SL), potentially SL 17:0;O/17:0;O (C_34_H_69_NO_6_S; Figure S4). Members of the chemoorganoheterotrophic *Bacteroidetes* phylum are the most common producers of SLs, where they play a key role in their gliding motility (Godchaux and Leadbetter 1988; Corcelli et al. 2004). Marine sediment is a typical habitat for *Bacteroidetes*, including methane‐rich sites such as the Guaymas Basin and the Hydrate Ridge, where they often thrive in proximity to *Beggiatoa* mats (Teske et al. 2002; Lanoil et al. 2001; Marchesi et al. 2001). Ramírez et al. (2021) recently detected *Bacteroidetes* in a neighbouring sediment core (5000‐11) via 16S rRNA gene Amplicon Sequence Variants, noting a comparable downward decrease with the highest abundance within the upper 7 cm of the core. The class‐level composition indicated a dominance of the anaerobic and fermenting members of the class *Bacteroidia*. Their anaerobic metabolism is consistent with the absence of SL within the discrete oxygen‐rich zone near the sediment–water interface.

Interestingly, the biosignatures represented by *m/z* 858.4658 and tentatively identified SL 17:0;O/17:0;O feature some spatial overlap within the suboxic zone (Figure 3). While *m/z* 858.4658 likely reflects sulphide‐oxidising bacteria or other oxygen‐ and/or nitrate‐dependent bacterial groups, the sulfonolipid rather indicates fermenting *Bacteroidetes*, possibly anaerobic *Bacteroidia*, given their absence in the top 2–3 mm and presence deeper in the reducing zone. Their partially overlapping spatial distribution underlines the colocalization of metabolically distinct microbial communities on small spatial scales. Not only is this type of microscale colocalization reminiscent of results from Wörmer et al. (2020), who applied a similar MSI approach to microbial mats, it also reinforces the observation of overlapping, metabolically distinct microbial populations in surficial Guaymas Basin sediment described by Engelen et al. (2021). This overlap, coupled with the observed sharp redox‐dependent transitions at ~2–3 mm and ~1.1 cm, underscores the intricate microbial community structure within just the top centimetre of the hydrothermal sediment.

### 3.4 Molecular Distribution Patterns in the Anoxic Sediment

**FIGURE 4:**
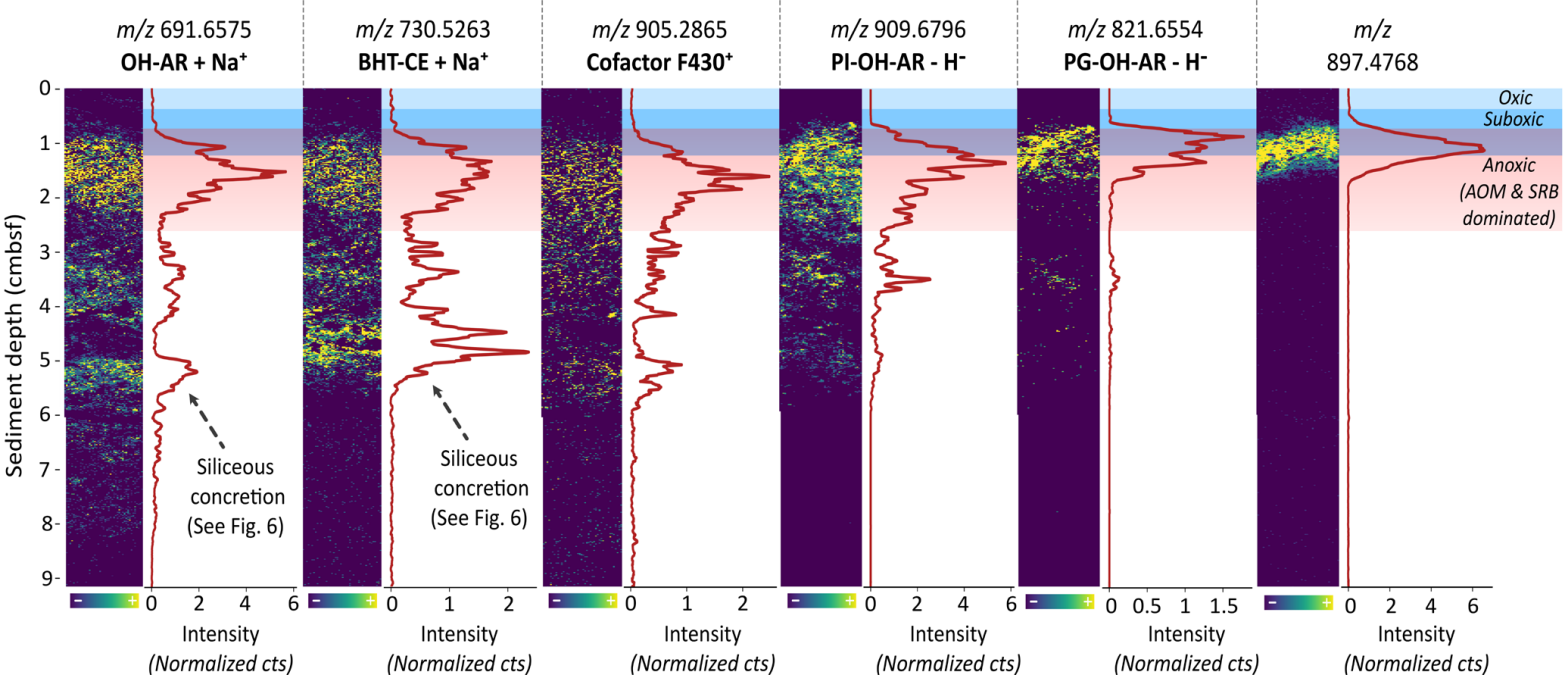
Spatial distribution of molecular features in the anoxic sediment zone of the Guaymas Basin. Ion intensity plots for six selected molecular features detected via MALDI‐MSI in positive (*m/z* 691.6575; *m/z* 730.5263; *m/z* 905.2865) and in negative ionisation mode (*m/z* 909.6796; *m/z* 821.6554; *m/z* 897.4768), showing distinct spatial patterns. The figure displays heatmaps for six molecular features contributing to NMF components with spatial patterns most dominant in the reducing zone. Line plots adjacent to each heatmap illustrate the sum of normalised counts for each 150‐μm depth interval, smoothed using a 5‐point rolling average. Colour gradients in the heatmaps range from blue (low intensity) to yellow (high intensity). Overlaid blue, cyan and red bars indicate the redox stratification of the sediment, with oxic, suboxic and anoxic (AOM and SRB dominated) zones labelled for reference. Compound identification is based on the respective *m/z* values combined with validation using complementary UHPLC/HRMS.

In total, 77 additional molecular features are associated with the transition to deeper, anoxic sediment layers (Figure 4 and Figure S5). Six features with high intensity were selected to cover a broad spectrum of lipid species, including the archaeal lipids *sn*‐2‐hydroxyarchaeol (OH‐AR), phosphatidylinositol hydroxyarchaeol (PI‐OH‐AR) and phosphatidylglycerol hydroxyarchaeol (PG‐OH‐AR), the bacterial lipid bacteriohopanetetrol cyclitol ether (BHT‐CE) and the archaeal nickel tetrapyrrole cofactor F430 (Figure 4). Molecular structures are provided in Figures S6 and S7. An unidentified compound at *m/z* 897.4768 was included because of its exceptionally well‐defined upper and lower boundaries, offering additional evidence for the distinct molecular zonation.

**FIGURE 5:**
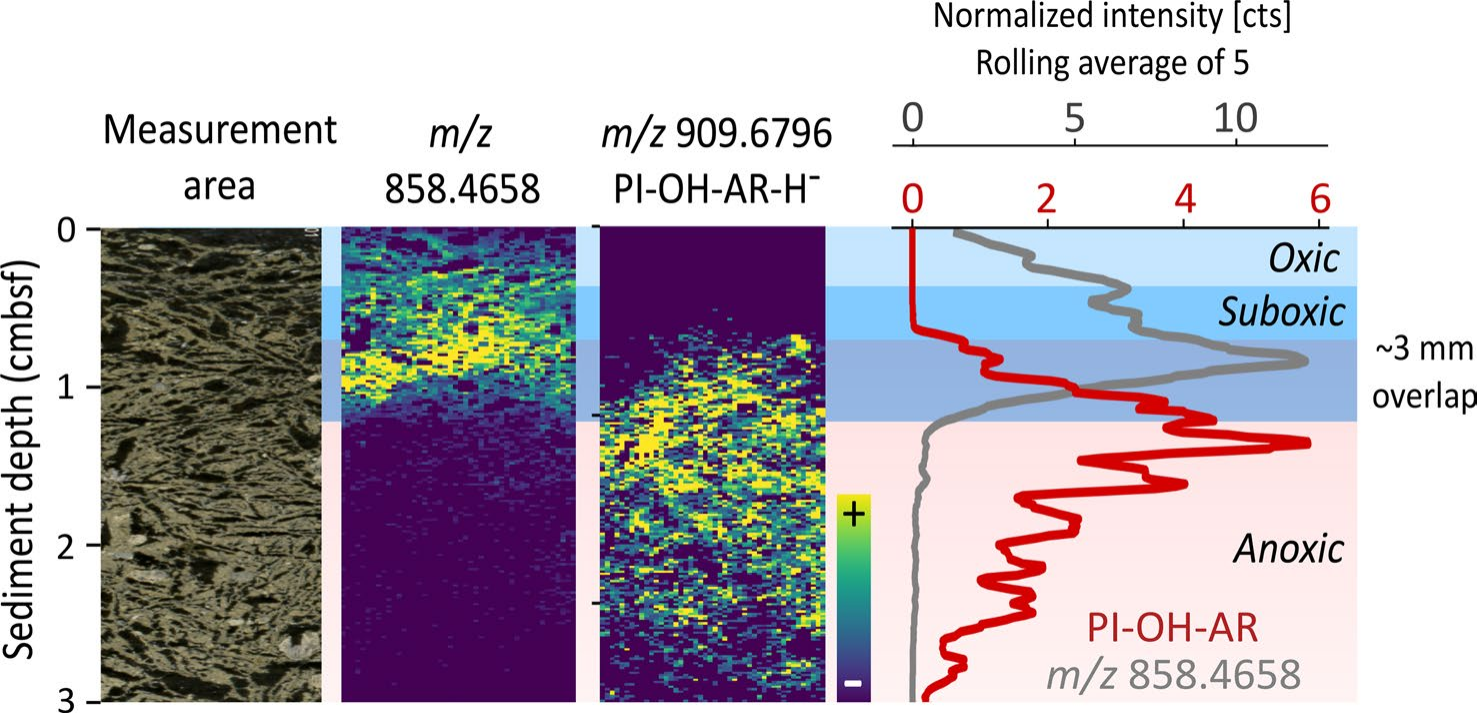
Zoomed‐in spatial distribution of molecular features *m/z* 858.4658 and PI‐OH‐AR‐H^‐^ in sediment core 5000‐9, highlighting the transition to the stable anoxic zone. Line plots adjacent to the heatmaps represent the normalised intensity profiles for each feature, smoothed using a moving average with a window of five. Both ions were detected in negative polarity while using a 1,5‐DAN matrix.

The sudden and pronounced appearance of all six molecular features in Figure 4 at approximately 1 cmbsf marks a striking transition and presumably reflects the onset of a stable anoxic zone. This transition is best illustrated by *m/z* 858.4658 and PI‐OH‐AR (Figure 5). When superimposing the downcore profiles of both compounds, a transition with minimal overlap spanning less than 3 mm becomes apparent, implying that two metabolically distinct microbial communities coexist within this transition zone.

The most prominent lipids within the anoxic zone are closely linked to anaerobic processes. OH‐AR, PI‐OH‐AR and PG‐OH‐AR are well‐established biomarkers for anaerobic, methane‐oxidising archaea (ANME), specifically of the ANME‐2 clade (Rossel et al. 2008) and have been reported repeatedly in methane‐rich hydrothermal environments (Hinrichs et al. 1999; Rossel et al. 2008, 2011). The vertical distribution is consistent with results from methyl‐coenzyme M reductase alpha subunit (*mcrA*) gene surveys and 16S rRNA analysis that placed ANME‐2 within surficial and temperate sediment layers (McKay et al. 2016; Ramírez et al. 2021; Biddle et al. 2012). Additional support for AOM is the presence of cofactor F430, the prosthetic group of the crucial enzyme MCR, which catalyses the methyl‐to‐methane and methane‐to‐methyl conversion in methanogenesis and methane oxidation, respectively (Mayr et al. 2008; Allen et al. 2014). Together, this biomarker inventory provides strong evidence that AOM mediated by ANME‐2 is concentrated within a distinct zone of ~2 cm, well separated from overlying aerobic processes, except for a minor overlap (~3 mm) at 1 cmbsf (Figure 5). While the upper boundary of the PI‐OH‐AR is sharp and well defined, the deeper transition is more diffuse, which may reflect the gradual onset of degradation during burial. PG‐OH‐AR likely exhibits a similar trend; however, the up to fourfold lower signal intensities of PG‐OH‐AR compared to PI‐OH‐AR hinder a confident description of its true depth extent. Similarly, observations on depth‐related trends in OH‐AR are compromised by its high abundance within the siliceous concretion embedded in the sediment (Figure 4).

Alongside typical AOM biomarkers, we detected a bacteriohopanepolyol (BHP) tentatively annotated as bacteriohopanetetrol cyclitol ether (BHT‐CE) based on characteristic fragmentation patterns in complementary UHPLC/HRMS data (Talbot et al. 2016; Hopmans et al. 2021) (Figure S6). Given the recent detection of unusual and structurally diverse hopanoids in a nearby hydrothermal sediment core (Mara et al. 2022), the labile and highly functional BHT‐CE may represent an important microbial precursor to those presumably thermally altered molecules. Despite BHT‐CE being ubiquitously detected in many environments (Talbot et al. 2003; Talbot and Farrimond 2007; Zhu et al. 2011), the distinct spatial distribution narrows down its potential microbial origin. The restricted occurrence of BHT‐CE in the anoxic zone strongly suggests an anaerobic microbial source, making the typical producers, such as aerobic methylotrophic and methanotrophic bacteria, unlikely contributors (Renoux and Rohmer 1985; Knani et al. 1994). Instead, *Geobacter* species are a probable source of BHT‐CEs in this setting. These anaerobic bacteria are particularly prevalent in anoxic, metal‐ and hydrocarbon‐rich environments (Holmes et al. 2002; Cummings et al. 2003), conditions strikingly similar to those found in the Guaymas Basin. Notably, BHT‐CE was found to be the major composite BHP synthesised by the dissimilatory metal‐ and sulphur‐reducing *Geobacter* *sulfurreducens* and *Geobacter* *metallireducens* (Eickhoff et al. 2013), supporting *Geobacter* spp. as a potential source of BHT‐CE in the anoxic Guaymas Basin sediment.

As complementary UHPLC/HRMS/MS spectra were not always available for all features, compound identification in MALDI‐MSI poses a significant challenge. Despite the high mass resolution and accuracy, many features, especially in the higher *m/z* range, remain elusive. One such feature is *m/z* 897.4768, which exhibits exceptionally high intensities between 1 and 2 cmbsf, with strikingly well‐defined upper and lower boundaries (Figure 4). The co‐occurrence of *m/z* 897.4768 with established AOM biomarkers, such as PG‐OH‐AR, suggests a strong link to ANME‐2 archaea or their associated syntrophic bacterial partners. The abrupt depletion of this molecule below and above this zone indicates a highly labile structure tightly linked to the spatial distribution of its producer.

Finally, the notable concentration of biosignatures within a thin zone of approximately 3 cm below the sediment–water interface, excluding those associated with the silica concretion, is most likely driven by the extreme thermal regime. Although we acknowledge the presence of active thermophilic microbial life deeper in the sediment (McKay et al. 2016; Ramírez et al. 2021), our results still highlight the temperate surface sediment (< 35°C) as the primary hotspot for microbial activity in this hydrothermally shaped ecosystem, consistent with cell count data (Meyer et al. 2013), lipid quantification (Schouten et al. 2003), dilution‐based cultivation studies (Teske et al. 2009) and DNA yield (Engelen et al. 2021).

Taken together, even within the uppermost 4 cm of the core, MALDI‐MSI revealed pronounced and sharply defined molecular transitions indicative of depth‐restricted, redox‐sensitive microbial processes within the sediment. These findings initially seem contradictory to the more gradual microbial shifts reported by Engelen et al. (2021), using a high‐resolution DGGE approach. However, as already emphasised by Engelen et al. (2021), hydrothermally impacted sediment in the Guaymas Basin exhibits a highly heterogeneous fine‐scale topography, where channels, fluid flow, or gas pockets hinder microbial communities from forming perfectly horizontal layers. In their DGGE approach, they sampled the entire cross‐section of the cores at 2‐mm intervals, integrating the information from a comparably large area. In contrast, MALDI‐MSI is performed on 100 μm‐thick slices, focusing on a much more localised scale. Accordingly, a shift that appears sharp in MALDI‐MSI may look gradual when averaged across the cross‐section of the core. This is further illustrated by the slightly tilted appearance of the molecular zonation, best observed in PI‐OH‐AR. Moreover, the silica concentration embedded in our sediment (Figure 1) may represent an additional contributing factor, as it could have locally slowed the hydrothermal flux, potentially allowing more distinct oxic, suboxic and anoxic zones to develop. In Engelen et al. (2021), no comparable structures were noted, allowing uninterrupted hydrothermal flow that may have obscured finer stratification. Thus, rather than contradictory, both datasets offer complementary insight into these highly heterogeneous environments.

### 3.5 Molecular Distribution Patterns in Silica Precipitate

**FIGURE 6:**
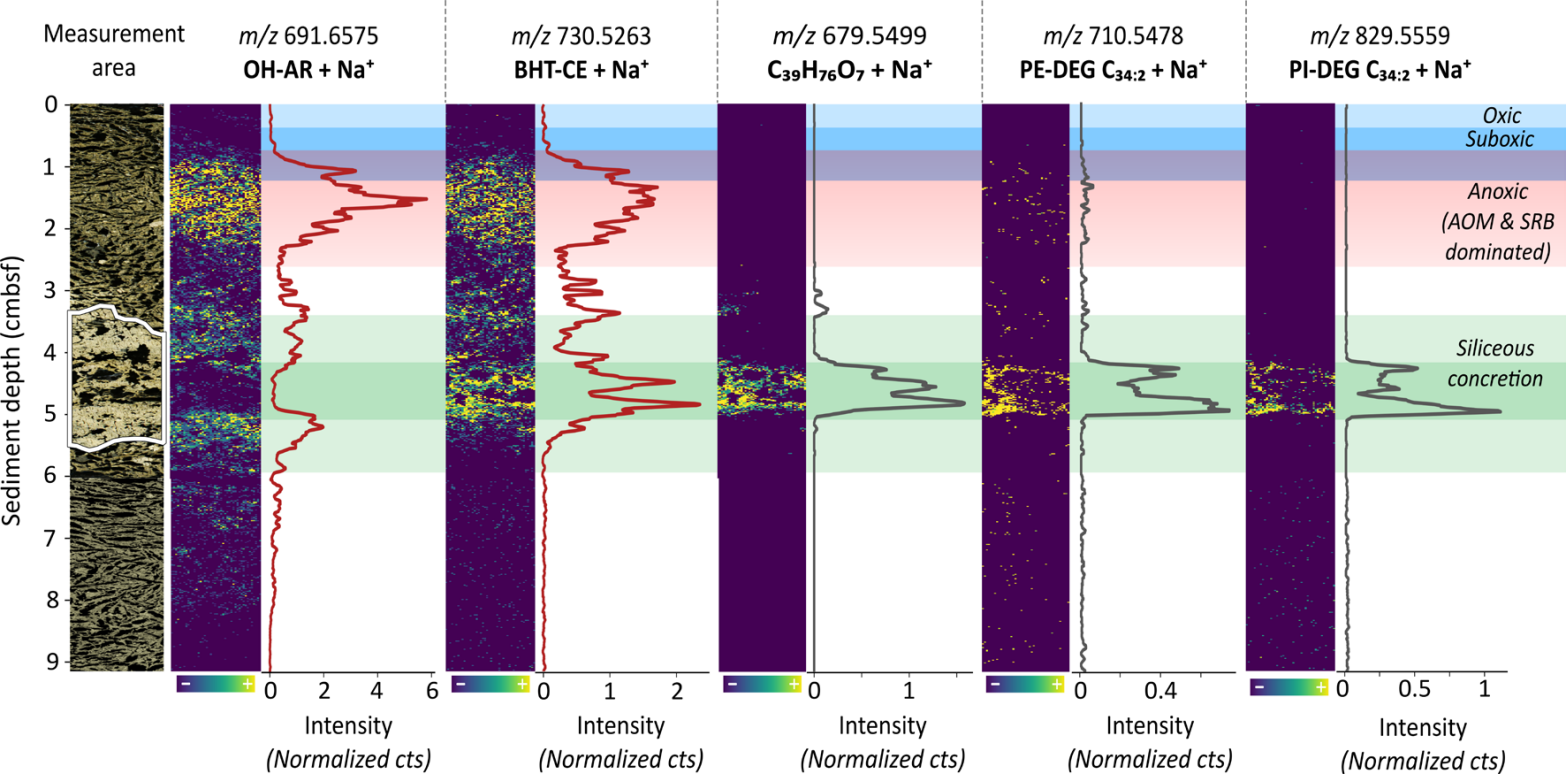
Spatial distribution of five molecular features associated with a silica concretion in the sediment of the Guaymas Basin (core 5000‐9). Ion intensity maps were acquired via MALDI‐MSI in positive polarity assisted by a 2,5‐DHB matrix. Location of silica concretion between approximately 3.5 and 5.5 cmbsf is indicated. Adjacent line plots represent the sum of normalised counts for each 75 μm sediment horizon, smoothed using a 5‐point rolling average. Colour gradients in the heatmaps range from blue (low intensity) to yellow (high intensity). Overlaid blue, cyan, red and green bars indicate the redox stratification of the sediment, with oxic, suboxic, anoxic (dominated by AOM and SRB) and silica concretion zones labelled for reference.

Intriguingly, several molecular signals were spatially associated with the silica concretion (Figure 6). While some lipids appear exclusively within the boundaries of the concretion, such as *m/z* 679.5499 (C_39_H_76_O_7_ + Na^+^) and the dietherglycerolipids PE‐DEG‐C_34:2_ and PI‐DEG‐C_34:2_, others extend beyond and are also abundant within the anoxic zone (1–3 cmbsf), best exemplified by BHT‐CE and OH‐AR. Lipid annotations are corroborated by complementary UHPLC/HRMS analysis of the isolated silica concretion, with additional silica‐related lipids presented in Figure S8 and Table S4.

It appears that the silica functions either as a hotspot for microbial colonisation distinct from microbial niches in the surrounding sediment or as a matrix that mediates the preservation of specific biomolecules. Intact polar lipids (IPLs) are prone to quick degradation, especially under high temperatures and hydrothermal influences that potentially accelerate the hydrolysis of the polar head group (White et al. 1979; Harvey et al. 1986). This ephemeral nature of IPLs under such conditions suggests that an active microbial community thrives in the ecological niche provided by the mineral matrix. Ether‐linked lipids of the type associated with the concretion have a long history as biomarkers for SRB, particularly, in hydrothermal and anoxic settings (Sturt et al. 2004; Rütters et al. 2001; Grossi et al. 2015). It is conceivable that these biomarkers reflect active SRB, fueled by downward advection of sulphate‐rich seawater into the porous silicate matrix via hydrothermal pumping or channelling. This is reminiscent of active endolithic ANME and SRB communities found in sediment‐hosted authigenic carbonates in various high methane‐flux sites (Marlow et al. 2014; Yanagawa et al. 2019; Parra et al. 2025). These findings demonstrate that mineral precipitates not only can archive past microbial activity but also support ongoing biochemical processes and host distinct microbial communities. Nevertheless, the pivotal role of silica sinters in preserving microbial lipids and recording biosignatures on geological time scales has been demonstrated repeatedly and must be considered as well (Sánchez‐García et al. 2023; Pancost et al. 2006; Teece et al. 2020). Thus, it is also plausible that the silica nodule represents a buried fragment once part of larger mineral structures near hydrothermal mounds that has entombed the lipid fingerprint of past microbial communities.

Interestingly, the archaeal lipid OH‐AR is most abundant within the outer rim of the silica precipitate, while the aforementioned bacterial lipids appear to accumulate within the centre (Figure 6). This phenomenon is corroborated by several additional archaeal lipids, such as AR and diglycosidic AR (2G‐AR), both valid biomarkers for ANME in this context (Rossel et al. 2008, 2011; Figure S5). While the environmental factors driving this pattern remain speculative, it might reflect current or past ecological niches fostering this spatial segregation of ANME and SRB that are now either preserved or active in the silica precipitate.

## 4 Conclusion

Performing MALDI‐MSI revealed a high degree of spatial stratification in the lipidome, characterised by sharp transitions linked to the steep redox gradients that unfold over millimetre scales. Leveraging the chemotaxonomic specificity of several annotated bacterial and archaeal lipids allowed us to infer the dominant microbial communities within each redox‐stratified zone. Notably, the sediment exhibits a tightly localised hotspot of lipids diagnostic for ANME‐2 archaea, confined to an ~2 cm‐thick interval within a stable anoxic zone (~1–3 cm below the seafloor), which sharply transitions into a near‐surface suboxic‐oxic zone, characterised by a distinctly different lipid inventory. Despite the presumed dynamic nature of the hydrothermal setting, these surprisingly sharp transitions suggest that key redox interfaces may be more stable than previously assumed and the associated microbial niches are strikingly persistent, perhaps supported by local mineralogical features within the sediment that moderate the hydrothermal flow.

## Author Contributions

**Janina Groninga:** conceptualization, visualization, writing – original draft, formal analysis, writing – review and editing, investigation. **Weimin Liu:** methodology, resources, writing – review and editing. **Jenny Altun:** methodology, resources. **Lars Wörmer:** ethodology, resources, writing – review and editing. **Andreas Teske:** resources, funding acquisition, writing – review and editing. **Kai‐Uwe Hinrichs:** conceptualization, funding acquisition, writing – review and editing, resources, supervision.

## Funding

This work was supported by the Deutsche Forschungsgemeinschaft (EXC‐2077‐390741603).

## Conflicts of Interest

The authors declare no conflicts of interest.

## Supporting information


**Table S1:** Measurement parameters for matrix‐assisted laser desorption ionisation mass spectrometry imaging (MALDI‐MSI) of the Guaymas Basin core 5000‐9.
**Table S2:** Total organic carbon and nitrogen content in Guaymas Basin core 5000‐9.
**Table S3:** List of lipids detected during MALDI‐MSI of Cathedral Hill sediment core 5000‐9.
**Table S4:**. List of lipids detected specifically within the silica precipitate.
**Figure S1:** Measurement area for an exemplary sediment slice (5000‐9; 0–6.1 cmbsf).
**Figure S2:** High‐resolution intensity maps (150 μm) from MALDI‐MSI for sediment core 5000‐9 (Downward decreasing spatial pattern).
**Figure S3:** High‐resolution intensity maps (150 μm) from MALDI‐MSI for sediment core 5000‐9 (Surface‐dominated signals).
**Figure S4:** High‐resolution intensity map (150 μm) of sulfonolipid SL (17:0;0/17:0;0).
**Figure S5:** High‐resolution intensity maps (150 μm) from MALDI‐MSI for sediment core 5000‐9 (AOM and SRB dominated zone).
**Figure S6:** High‐resolution intensity map (150 μm) of bacteriohopanetetrol cyclitol ether.
**Figure S7:** Selection of molecular features identified using MALDI‐MSI on sediment core 5000‐9.
**Figure S8:** High‐resolution intensity maps (150 μm) from MALDI‐MSI for sediment core 5000‐9 (Silica concretion).

## Data Availability

The data that support the findings of this study are openly available in Zenodo at https://doi.org/10.5281/zenodo.16903250, reference number 10.5281/zenodo.16903250.
