# Supplementary material for: Millimetre‐Scale Stratification of Microbial Communities in Hydrothermal Sediments
Source: Environ Microbiol. 2026 Jan 4;28(1):e70227. doi: 10.1111/1462-2920.70227 (PMC12766260; doi:10.1111/1462-2920.70227)
Supplement: Supplementary file 1 — Table S1: Measurement parameters for matrix‐assisted laser desorption ionisation mass spectrometry imaging (MALDI‐MSI) of the Guaymas Basin core 5000‐9. Table S2: Total organic carbon and nitrogen content in Guaymas Basin core 5000‐9. Table S3: List of lipids detected during MALDI‐MSI of Cathedral Hill sediment core 5000‐9. Table S4:. List of lipids detected specifically within the silica precipitate. Figure S1: Measurement area for an exemplary sediment slice (5000‐9; 0–6.1 cmbsf). Figure S2: High‐resolution intensity maps (150 μm) from MALDI‐MSI for sediment core 5000‐9 (Downward decreasing spatial pattern). Figure S3: High‐resolution intensity maps (150 μm) from MALDI‐MSI for sediment core 5000‐9 (Surface‐dominated signals). Figure S4: High‐resolution intensity map (150 μm) of sulfonolipid SL (17:0;0/17:0;0). Figure S5: High‐resolution intensity maps (150 μm) from MALDI‐MSI for sediment core 5000‐9 (AOM and SRB dominated zone). Figure S6: High‐resolution intensity map (150 μm) of bacteriohopanetetrol cyclitol ether. Figure S7: Selection of molecular features identified using MALDI‐MSI on sediment core 5000‐9. Figure S8: High‐resolution intensity maps (150 μm) from MALDI‐MSI for sediment core 5000‐9 (Silica concretion). [file EMI-28-e70227-s001.pdf]

Supplementary information for **Millimeter-scale stratification of microbial communities in hydrothermal sediments**

Janina Groninga[1,2*], Weimin Liu[1], Lars Wörmer[1], Jenny Altun[1,2], Andreas Teske[3], Kai-Uwe Hinrichs[1,2]

[1]MARUM – Center for Marine Environmental Sciences, University of Bremen, 28359 Bremen, Germany

[2]Faculty of Geosciences, University of Bremen, 28359 Bremen, Germany

[3]Department Earth, Marine and Environmental Sciences, University of North Carolina at Chapel Hill, Chapel Hill, NC 27599-3300, USA

[*]**Corresponding author: E-mail:** jgroninga@marum.de

This PDF file includes:

Supplementary Figures S1 to S8

Supplementary Tables S1 to S4

Supplementary Table S1. Measurement parameters for matrix-assisted laser desorption ionization mass spectrometry imaging (MALDI-MSI) of the Guaymas Basin core 5000-9 (0 - 11.5 cmbsf). The top panel displays parameters for measurements using a 2,5-dihydroxybenzoic acid (2,5-DHB) matrix, while the bottom panel presents parameters for measurement assisted by a 1,5-diaminonaphthalene (1,5-DAN) matrix. Included are the first quadrupole (Q1) mass, the Q1 isolation window, laser power, number of laser shots per spot, laser frequency (Hz), Time-of-flight, polarity, collision voltage (in V), the data acquisition size, chosen lock mass calibrants, dominated by matrix oligomers; per CASI window (*m/z* and chemical formula); the percentage of successful spectra calibration, and the number of spectra recorded for each measurement area.

	Depth [cm]	Q1 Mass [m/z]	Q1 Isolation Window	Laser Power	Laser Shots	Laser Frequency [Hz]	Time of Flight [ms]	Polarity	Collision Voltage [V]	Data Acquisition size	Lock Mass Calibrant Chemical Formula	Lock Mass Calibrant *m/z*	% calibrated	No. Spectra
**2,5-DHB matrix**	0-6.1	250	200	75	300	350	0.6	positive	-5	150-2000-1M	$C_7H_4O_3Na$; $C_7H_6O_4Na$	159.00527, 177.01583	99	17595
	6.1-11.5	250	200	75	300	350	0.6	positive	-5	150-2000-1M	$C_7H_4O_3Na$; $C_7H_6O_4Na$	159.00527, 177.01583	99.7	16183
	0-6.1	385	150	65	250	250	0.7	positive	-5	150-2000-1M	$C_{14}H_{10}O_7Na$; $C_{21}H_{14}O_{10}Na$; $C_{14}H_{12}O_8Na$	313.03187; 449.04792; 331.04244	99.5	18630
	6.1-11.5	385	150	65	250	250	0.7	positive	-5	150-2000-1M	$C_{14}H_{10}O_7Na$; $C_{21}H_{14}O_{10}Na$; $C_{14}H_{12}O_8Na$	313.03187; 449.04792; 331.04244	99.4	16183
	0-6.1	535	150	63	250	250	0.7	positive	-5	150-2000-1M	$C_{28}H_{18}O_{13}Na$; $C_{21}H_{16}O_{11}Na$	585.06396, 467.05848	88.1	18630
	6.1-11.5	535	150	68	250	250	0.7	positive	-5	150-2000-1M	$C_{28}H_{18}O_{13}Na$; $C_{21}H_{16}O_{11}Na$	585.06396, 467.05848	97.6	16183
	0-6.1	700	200	78	250	500	0.8	positive	-10	600-2000 256k	$C_{35}H_{22}O_{16}Na$	721.08001	93.7	18630
	6.1-11.5	700	200	76	250	500	0.8	positive	-10	600-2000 256k	$C_{35}H_{22}O_{16}Na$	721.08001	85.5	16192
	0-6.1	900	210	78	250	250	1.1	positive	-10	600-2000 256k	$C_{42}H_{26}O_{19}Na$	857.09605	91.8	18630
	6.1-11.5	900	200	80	250	250	1.1	positive	-10	600-2000 256k	$C_{42}H_{26}O_{19}Na$	857.09605	83.5	16192
**1,5-DAN matrix**	0-6.1	230	150	75	300	350	0.5	negative	5	150-2000-1M	$C_{10}H_9N_2$	157.07712	100	17460
	6.1-11.5	230	150	75	300	350	0.5	negative	5	150-2000-1M	$C_{10}H_9N_2$	157.07712	100	15953
	0-6.1	385	150	70	300	350	0.5	negative	5	150-2000-1M	$C_{30}H_{23}N_5$	453.19589	100	17460
	6.1-11.5	385	150	70	300	350	0.5	negative	5	150-2000-1M	$C_{30}H_{23}N_5$	453.19589	100	15953
	0-6.1	535	150	60	250	250	0.7	negative	5	150-2000-1M	$C_{30}H_{25}N_6$	469.21462	100	18864
	6.1-11.5	535	150	65	250	250	0.7	negative	5	150-2000-1M	$C_{30}H_{25}N_6$	469.21462	100	16368
	0-6.1	700	210	67	500	700	0.8	negative	10	600-2000 256k	$C_{40}H_{31}N_7$	609.26464	99.7	18330
	6.1-11.5	700	210	63	500	300	0.8	negative	10	600-2000 256k	$C_{40}H_{31}N_7$	609.26464	100	16368
	0-6.1	900	210	83	700	500	1.1	negative	10	600-2000 256k	$C_{60}H_{41}N_{11}$	915.35519	97.8	18630
	6.1-11.5	900	210	90	500	700	1.1	negative	10	600-2000 256k	$C_{62}H_{28}N_6$; $C_{67}H_{42}N_6$	856.23809, 930.3476	99.2	16470

Supplementary Table S2. Total organic carbon and nitrogen content in Guaymas Basin core 5000-9, with respective $\delta^{13}C$ values (in ‰ vs. VPDB), $\delta^{15}N$ values (in ‰ vs. air N_2), and C/N ratio.

Sediment depth [cm]	TOC [wt%]	$\delta^{13}C$ TOC [‰]	TN [wt%]	$\delta^{15}N$ [‰]	C/N
0-1	4.0	-23.6	0.3	7.5	13.8
1-2	4.4	-24.0	0.3	9.5	14.9
2-3	4.8	-23.2	0.3	10.1	18.0
3-4	3.9	-22.6	0.3	9.2	16.9
4-5	3.4	-21.8	0.2	9.2	17.5
5-6	3.7	-21.9	0.2	9.3	18.4
6-7	3.2	-21.6	0.2	9.0	19.2
7-8	3.5	-22.2	0.2	8.9	21.2
8-9	3.0	-21.8	0.2	7.2	19.5
9-10	2.3	-21.6	0.1	7.5	21.6
10-11	2.2	-22.2	0.1	7.5	25.4
11-12	1.8	-21.2	0.1	4.9	18.9
12-13	1.7	-21.0	0.1	6.7	19.5
13-14	1.8	-21.0	0.1	5.5	20.7
14-15	2.7	-23.3	0.1	7.6	33.3
15-16	2.9	-22.5	0.1	7.4	25.0
16-17	2.2	-21.5	0.1	8.0	24.1
17-18	2.4	-	0.1	4.0	27.7
18-19	2.5	-	0.1	4.2	25.3
19-20	2.4	-22.7	0.1	4.3	22.2

Supplementary Table S3. List of lipids detected during MALDI-MSI of Cathedral Hill sediment core 5000-9, with measured and theoretical *m/z*, the corresponding ion species, the proposed chemical formula and compound name.

**MALDI-MSI**		**Ion species**	**Proposed Chemical Formula**	**Name**
m/z (meas.)	***m/z (theo.)***			
330.3596	330.359123	$[M+H]^+$	$C_{18}H_{43}N_5$	uncharacterized polyamine
535.2715	535.270367	$[M+H]^+$	$C_{33}H_{34}N_4O_3$	Pyropheophorbide *a*
617.1853	617.184624	$[M+H]^+$	$C_{34}H_{32}FeN_4O_4$	Heme
618.4715	618.476187	$[M-H]^-$	$C_{34}H_{69}NO_6S$	SL (17:0;0/17:0;0)
675.6628	675.662567	$[M+Na]^+$	$C_{43}H_{88}O_3$	AR
691.6575	691.657482	$[M+Na]^+$	$C_{43}H_{88}O_4$	OH-AR
730.5263	730.522839	$[M+Na]^+$	$C_{41}H_{73}NO_8$	BHT-CE
813.5661	813.567719	$[M+H]^+$	$C_{53}H_{72}N_4O_3$	Pyropheophytin *a*
821.6554	821.662998	$[M-H]^-$	$C_{46}H_{95}O_9P$	PG-OH-AR
893.6788	893.684127	$[M-H]^-$	$C_{49}H_{99}O_{11}P$	PI-AR
905.2865	905.286205	$[M]^+$	$C_{42}H_{51}N_6NiO_{13}$	Cofactor F430
909.6796	909.679042	$[M-H]^-$	$C_{49}H_{99}O_{12}P$	PI-OH-AR
999.7673	999.768214	$[M+Na]^+$	$C_{55}H_{108}O_{13}$	2G-AR

Supplementary Table S4. List of lipids detected specifically within the silica precipitate in addition to those shown in Fig. 7. The table includes the corresponding measured and theoretical *m/z* values for the respective ion species, along with the proposed chemical formulas and compound names, if identified.

MALDI-MSI		Ion species	Proposed Chemical Formula	Name
m/z (meas.)	*m/z (theo.)*			
651.5188	651.517025	$[M+Na]^+$	$C_{37}H_{72}O_7$	unknown
667.5478	667.548325	$[M+Na]^+$	$C_{38}H_{76}O_7$	unknown
679.5499	679.548325	$[M+Na]^+$	$C_{39}H_{76}O_7$	unknown
691.5489	691.548325	$[M+Na]^+$	$C_{40}H_{76}O_7$	unknown
710.5478	710.545897	$[M+Na]^+$	$C_{39}H_{78}NO_6P$	PE-DEG-$C_{34:2}$
724.5274	724.525161	$[M+Na]^+$	$C_{39}H_{76}NO_7P$	PE-AEG-$C_{34:2}$
742.5382	742.535726	$[M+Na]^+$	$C_{39}H_{78}NO_8P$	PG-Cer-$C_{36:0}$
807.5728	807.574577	$[M-H]^-$	$C_{43}H_{85}O_{11}P$	PI-DEG-$C_{34:1}$
809.585	809.590227	$[M-H]^-$	$C_{43}H_{87}O_{11}P$	PI-DEG-$C_{34:0}$
821.5491	821.553841	$[M-H]^-$	$C_{43}H_{83}O_{12}P$	PI-AEG-$C_{34:1}$
829.5559	829.556521	$[M+Na]^+$	$C_{43}H_{83}O_{11}P$	PI-DEG-$C_{34:2}$
833.5861	833.590227	$[M-H]^-$	$C_{45}H_{87}O_{11}P$	PI-DEG-$C_{36:2}$

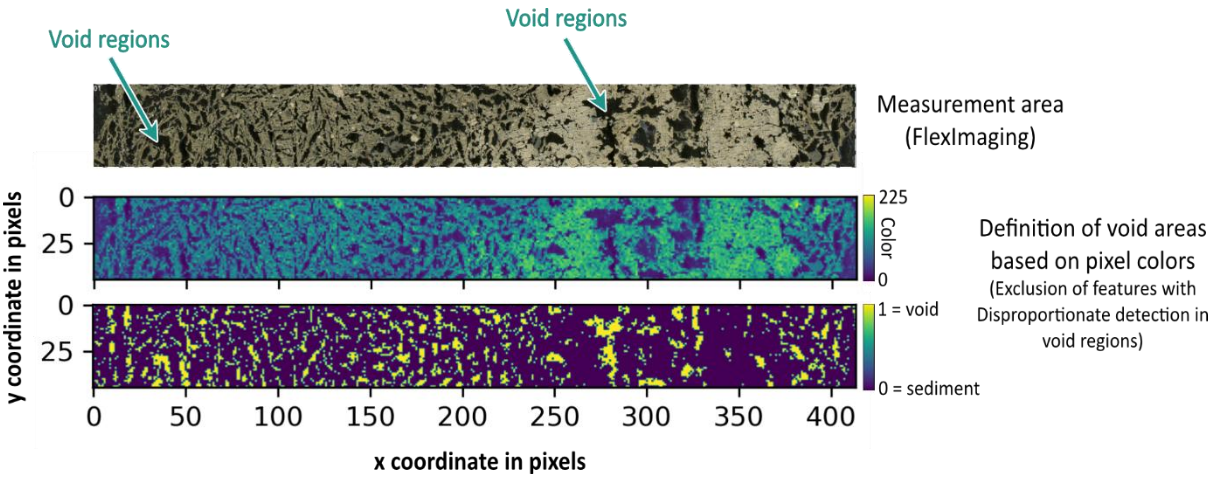

Supplementary Figure S1. Measurement area for an exemplary sediment slice (5000-9; 0 - 6.1 cmbsf) exported from FlexImaging. The sediment contains several void regions, remnants of freezing and sample preparation, indicated in the top panel. The middle panel shows a color transformation of the core image used to identify coordinates associated with void regions. The bottom panel highlights the corresponding void regions in yellow. Molecular features disproportionately represented within these void regions were excluded from NMF decomposition. Specific thresholds varied by sample. Generally, features for which more than 50% of the total intensity across the sample area originated from void regions were excluded.

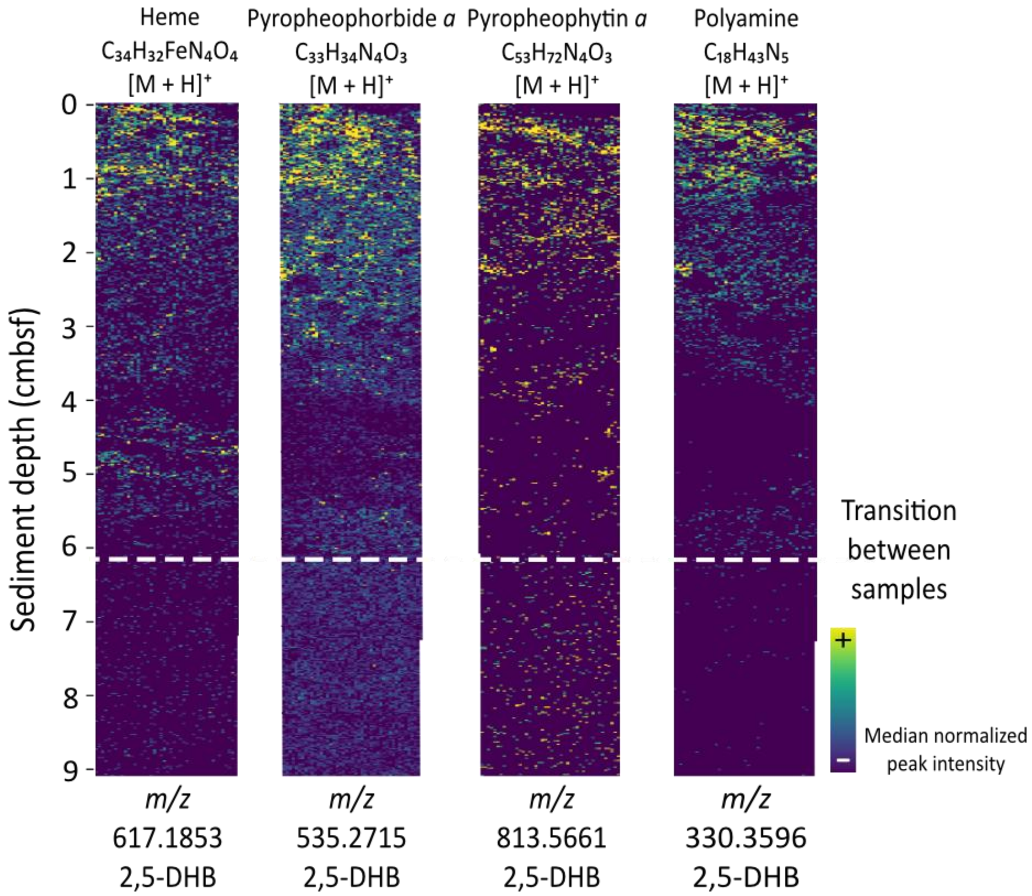

Supplementary Figure S2. High-resolution intensity maps (150 µm) from MALDI-MSI for sediment core 5000-9, zoomed into the top 9 cmbsf. Each panel displays gradually downward decreasing spatial distribution of a specific molecular feature including heme, pyropheophorbide *a*, pyropheophytin *a* and a not further specified polyamine. Intensity is color-coded, with purple indicating low signal intensity and yellow representing high signal intensity. The corresponding *m/z* and the MALDI-matrix that facilitated detection are indicated below.

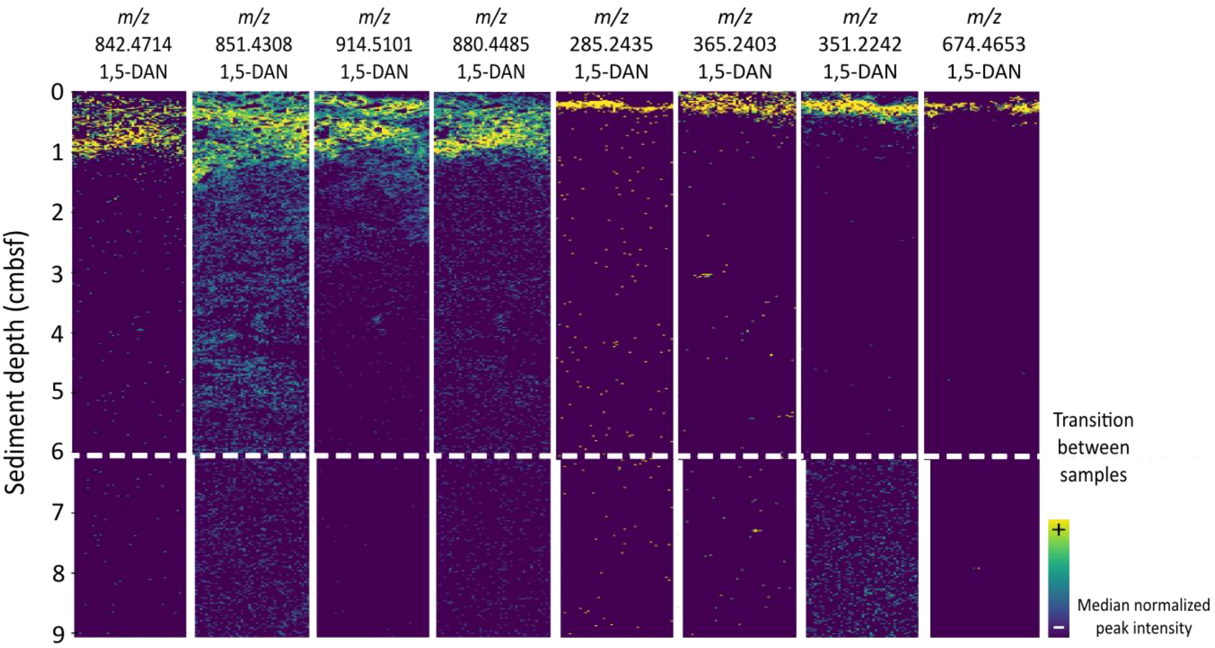

Supplementary Figure S3. High-resolution intensity maps (150 µm) from MALDI-MSI for sediment core 5000-9, zoomed into the top 9 cmbsf. Each panel displays the surface-dominated spatial distribution of specific molecular features, shown as the measured *m/z*. Confident identification of the shown signals was not feasible. The MALDI-matrix that facilitated detection is indicated. The intensity is color-coded, with purple indicating low signal intensity and yellow representing high signal intensity.

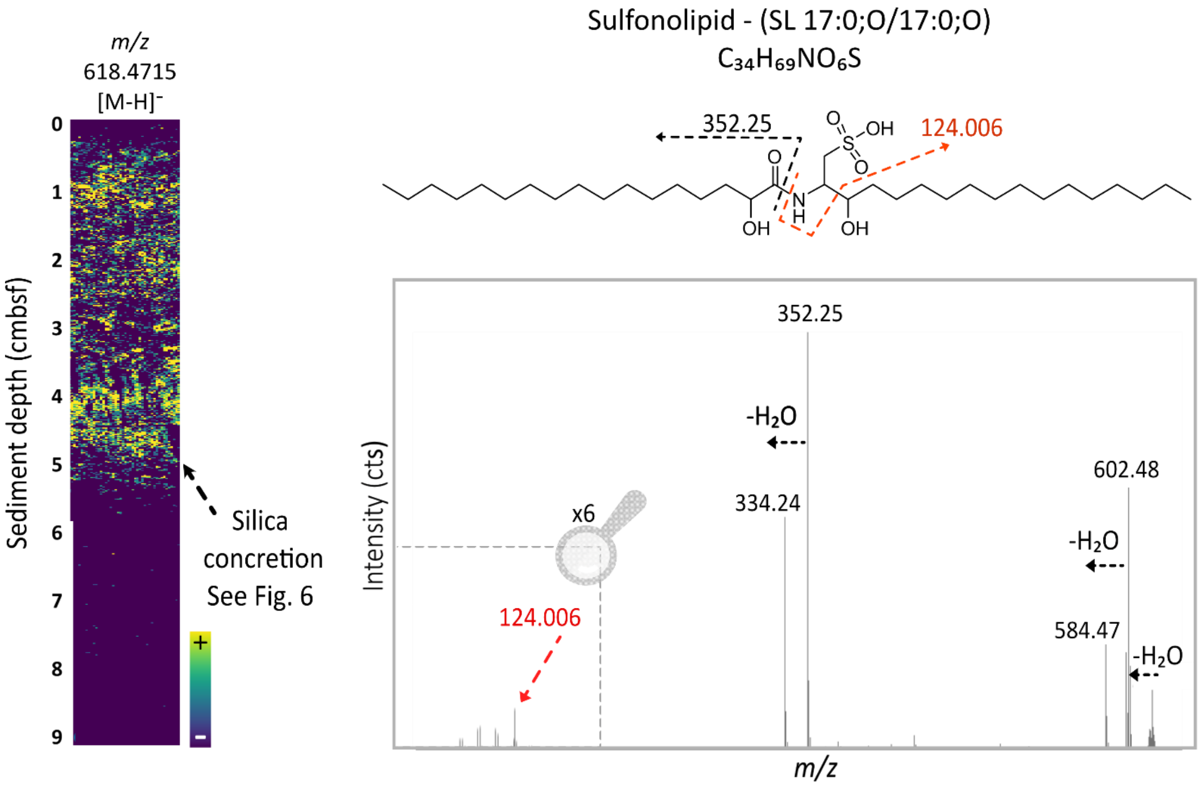

Supplementary Figure S4. High-resolution intensity map (150 µm) of sulfonolipid SL (17:0;0/17:0;0) yielded from MALDI-MSI, displaying the spatial distribution within sediment core 5000-9. The right panel shows a representative MS/MS spectrum, which facilitated robust structural identification utilizing the characteristic fragmentation pattern, with emphasis on the characteristic sulfonic acid head group ion (*m/z* 124.00).

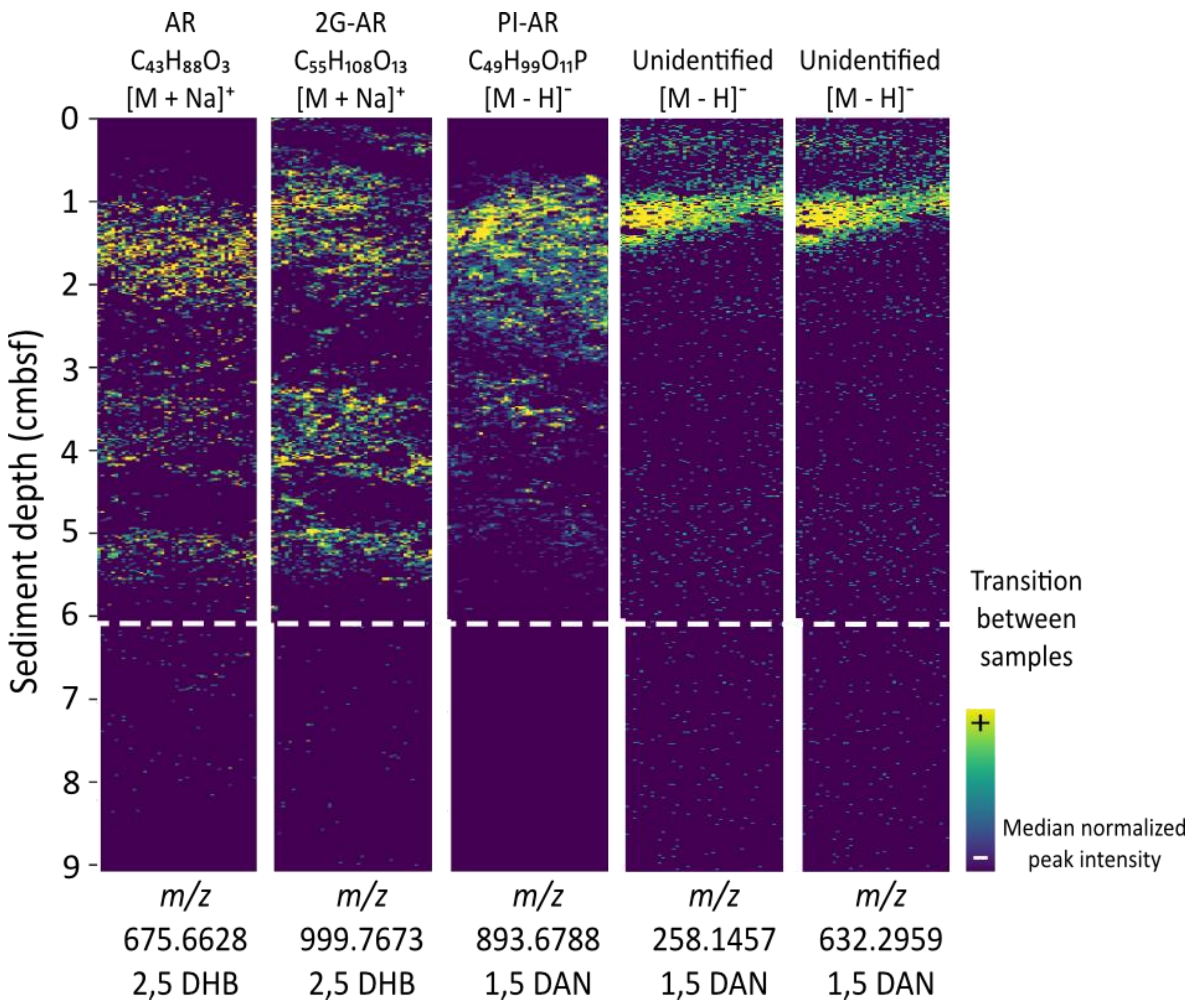

Supplementary Figure S5. High-resolution intensity maps (150 µm) from MALDI-MSI for sediment core 5000-9, zoomed into the top 9 cmbsf. Each panel represents the spatial distribution of molecular features, including archaeol (AR), diglycosidic archaeol (2G-AR), and phosphatidylinositol archaeol (PI-AR), along with two yet unidentified *m/z* values. The corresponding *m/z* and the MALDI-matrix that facilitated detection are indicated below. The intensity is color-coded, with purple indicating low signal intensity and yellow representing high intensity. Signal intensity is concentrated between 1 and 3 cmbsf, corresponding to the reducing zone of the core, with some detection of AR and 2G-AR within the siliceous concretion between 3.5 and 5.5 cmbsf.

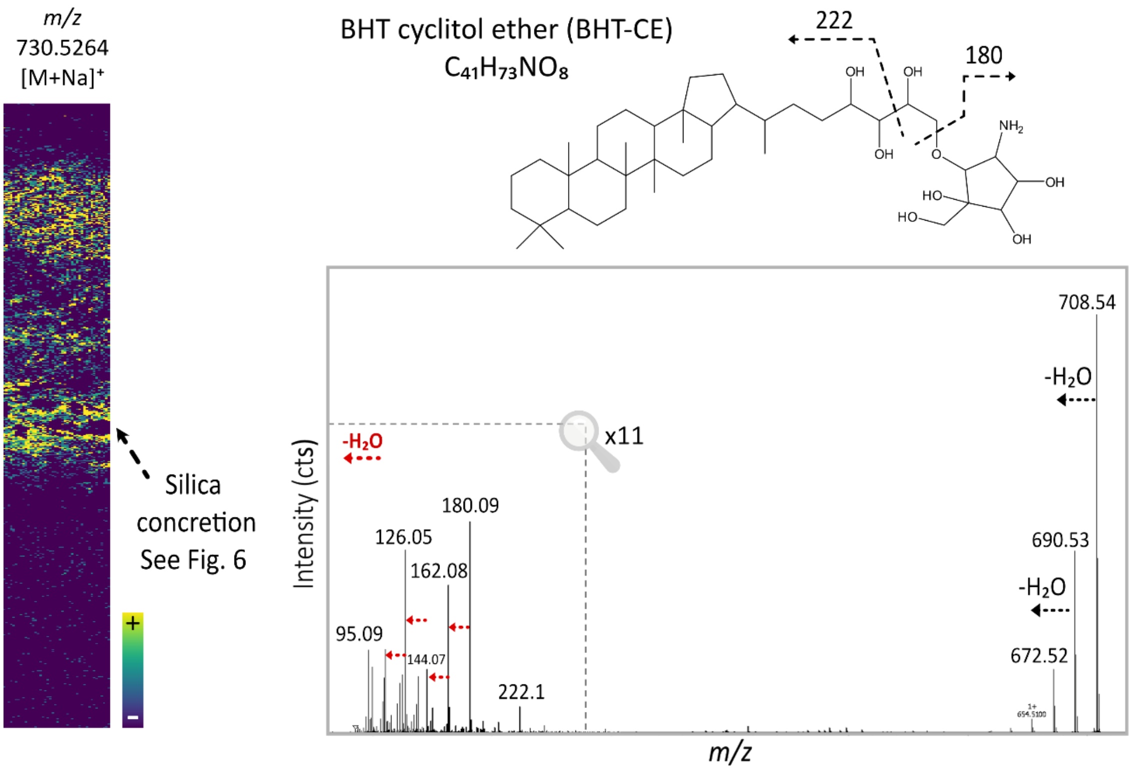

Supplementary Figure S6. High-resolution intensity map (150 µm) of bacteriohopanetetrol cyclitol ether (BHT-CE) yielded from MALDI-MSI, displaying the spatial distribution within sediment core 5000-9. The right panel shows a representative MS/MS spectrum, which facilitated robust structural identification utilizing the characteristic fragmentation pattern described by Talbot et al. 2016 and Hopmans et al. 2021.

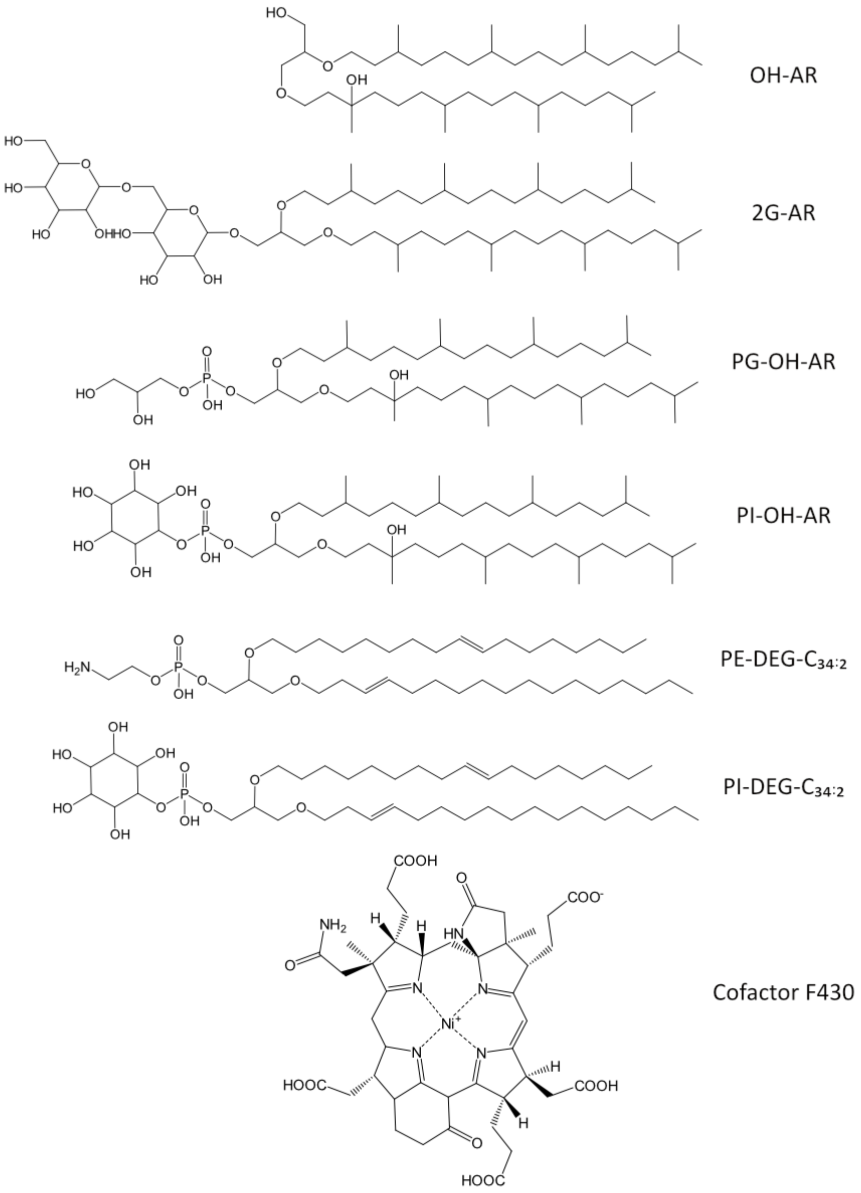

Supplementary Figure S7. Selection of molecular features identified using MALDI-MSI on sediment core 5000-9. The displayed features include hydroxy archaeol (OH-AR), diglycosidic archaeol (2G-AR), phosphatidylglycerol hydroxy archaeol (PG-OH-AR), phosphatidylinositol hydroxy archaeol (PI-OH-AR), phosphatidylethanolamine diether $C_{34:2}$ (PE-DEG-$C_{34:2}$), phosphatidylinositol diether $C_{34:2}$ (PI-DEG-$C_{34:2}$), and Cofactor F430.

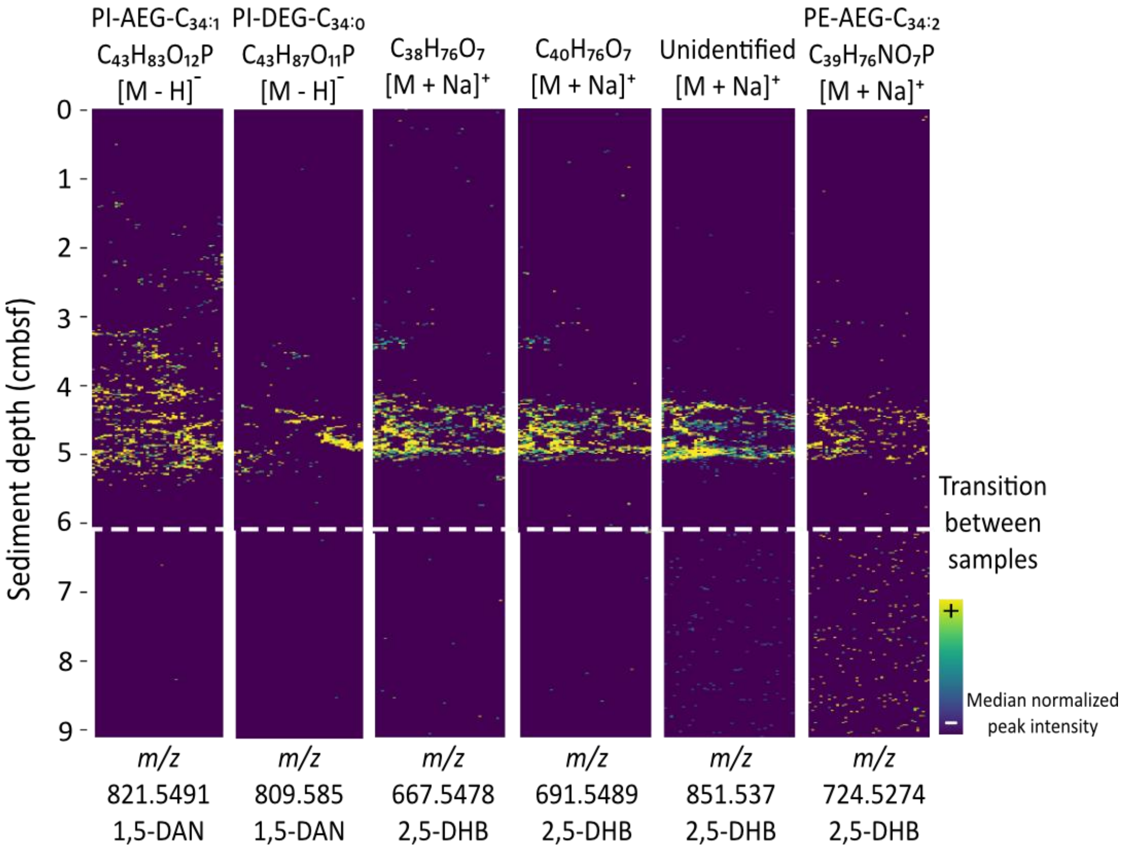

Supplementary Figure S8. High-resolution intensity maps (150 µm) from MALDI-MSI for sediment core 5000-9, zoomed into the top 9 cmbsf. Each panel represents the spatial distribution of molecular features, including phosphatidylinositol acyl ether glycerol $C_{34:1}$ (PI-AEG-$C_{34:1}$), phosphatidylinositol diether $C_{34:0}$ (PI-DEG-$C_{34:0}$), phosphatidylethanolamine acyl ether glycerol $C_{34:2}$ (PE-AEG-$C_{34:2}$), two *m/z* values tentatively annotated as $C_{38}H_{76}O_7$ and $C_{40}H_{76}O_7$, along with one yet unidentified *m/z* value. The corresponding *m/z* and the MALDI-matrix that facilitated detection are indicated below. The intensity is color-coded, with purple indicating low signal intensity and yellow representing high intensity. Signal intensity is concentrated between 4 and 5 cmbsf, corresponding to the siliceous concretion embedded within the sediment.
